# Reproductive Health Communication in Spanish: A Standardized Patient-Based Module for Second-Year Medical Students

**DOI:** 10.15766/mep_2374-8265.11635

**Published:** 2026-09-08

**Authors:** Alexandra López Vera, Jessica Lin, Jocelyn Sagahon-Ramirez, Daniel Ninan

**Affiliations:** 1 Assistant Professor, Department of Medical Education, California University of Science and Medicine; 2 Third-Year Medical Student, California University of Science and Medicine; 3 Third-Year Medical Student, California University of Science and Medicine; 4 Third-Year Medical Student, California University of Science and Medicine

**Keywords:** Medical Spanish, OSCE, Clinical Communication, Reproductive Health, Simulation-Based Education, Language Concordance, Task-Based Learning, Clinical Skills Assessment, Standardized Patient

## Abstract

**Introduction:**

Spanish-speaking patients with non-English language preferences experience communication inequities, particularly in encounters that are sensitive and require nuanced language. Prior medical Spanish curricula have improved general communication skills but have largely focused on other organ systems, with limited attention to reproductive health. To address this gap, we developed a 3-week reproductive medical Spanish module focused on reproductive health communication in Spanish.

**Methods:**

We implemented a 3-week module for second-year medical students that included faculty-led sessions, small-group activities, and standardized patient encounters. Assessment consisted of a 12-minute OSCE using a standardized patient case of a 30-year-old with lower abdominal pain requiring a focused reproductive history and patient-centered communication in Spanish. Performance was evaluated using an observational rubric with published validity evidence for assessing medical Spanish oral proficiency in standardized patient encounters.

**Results:**

A total of 128 students completed the final assessment. The mean score was 77.42% (*SD* = 2.28), with a median of 78% (range 68%-92%). More than half (53.9%) scored 80% or higher, and 87.5% scored at least 70%, demonstrating the ability to obtain a focused reproductive health history and communicate effectively in Spanish during a standardized patient encounter.

**Discussion:**

Learners completed core reproductive health communication tasks in Spanish during a standardized patient encounter. The module addressed an underrepresented area in medical Spanish education and demonstrated the feasibility of integrating reproductive health communication training within a systems-based curriculum.

## Educational Objectives

By the end of this activity, learners will be able to:

1. Conduct a focused reproductive health history in Spanish by eliciting the chief complaint and relevant symptom characteristics.
2. Apply appropriate reproductive health vocabulary and grammar in Spanish to describe menstrual, pregnancy-related, and other reproductive symptoms.
3. Demonstrate patient-centered communication in Spanish, including establishing rapport, ensuring comfort and privacy, and using respectful, inclusive, nonassumptive language.

## Introduction

Language is central to safe, patient-centered clinical care, yet communication inequities persist for patients with non-English language preferences (NELP).^1^ Spanish-speaking patients experience disparities in reproductive, prenatal, and postpartum care, including reduced comprehension and worse outcomes.^2-5^ These inequities are linked to structural communication barriers, as limited English proficiency is associated with lower-quality contraceptive counseling, decreased understanding of reproductive symptoms, and increased risk of adverse pregnancy experiences.^3,6,7^ These challenges highlight a gap in training medical students to conduct sensitive reproductive health encounters in Spanish.

Trained interpreters are essential for patients with NELP. Reproductive health encounters are sensitive and time-pressured, requiring nuanced, patient-centered communication.^8,9^ These features underscore the need to prepare trainees for language-concordant communication.

Existing medical Spanish programs improve vocabulary and communication confidence.^10,11^ Standardized patient (SP) encounters have supported experiential learning in this context.^12^ However, prior *MedEdPORTAL* publications have focused on musculoskeletal, dermatologic, or endocrine communication, leaving a gap in reproductive health language training.^13,14^ To our knowledge, no published *MedEdPORTAL* curriculum describes a structured, evaluated reproductive medical Spanish module that integrates anatomy, pregnancy, and communication during physical examinations through SP encounters.

The target audience was second-year medical students in a required, systems-based medical Spanish curriculum. At this stage, learners had foundational clinical Spanish skills but lacked training in reproductive health communication. Needs assessment data and faculty observations identified low confidence, inconsistent terminology use, and difficulty navigating sensitive conversations.

To address this gap, we developed a 3-week reproductive medical Spanish module aligned with the reproductive system block. The module integrated instructor-led sessions, guided communication practice, and SP encounters to support skill development, with a final SP-based OSCE used as the primary performance-based assessment.

The module's design was informed by established educational principles. It drew on task-based language teaching,^15^ which emphasizes authentic, communicative tasks, and backward design,^16^ which aligns objectives, instruction, and assessment. Consistent with experiential and deliberate-practice principles, learners applied skills in realistic SP encounters with structured debriefing and engaged in repeated, progressively complex practice with immediate feedback; the small-group format further supported collaborative, peer-based learning. The module was designed to build Spanish-language and patient-centered communication skills needed to conduct reproductive health encounters; it was not intended to teach clinical reasoning, differential diagnosis, or disease management, which are addressed in the concurrent reproductive system block of the Doctor of Medicine (MD) curriculum. Clinical content embedded in the cases was included to create realistic communication contexts and to reinforce—rather than introduce—material taught in that block.

## Methods

### Activity Design and Development

To ensure clinical accuracy and alignment with evidence-based practice, each SP case specifies a working diagnosis and includes a brief, facilitator-facing clinical background summarizing the evidence-based history, examination, and assessment relevant to the chief complaint and its leading differential diagnoses. All cases and supporting materials—including the history-taking questions, clinical workup, and patient communication—were reviewed by a family medicine physician, whose scope of practice encompasses the outpatient reproductive health presentations depicted, and were cross-checked against the content of the institutional reproductive system block prior to implementation.

### Curricular Context and Learner Preparation

The Vida Medical Spanish program at the California University of Science and Medicine (CUSM) is a required component of the preclerkship curriculum and is integrated within the systems-based structure of the MD program. As part of this longitudinal curriculum, we developed a 3-week medical Spanish reproductive system module for second-year medical students, building on prior modules implemented within the Vida Medical Spanish program.^14,17^

A total of 131 second-year medical students participated in the module. Before entering the reproductive module, students had completed the mandatory first-year medical Spanish curriculum. No additional facilitator training was required because sessions were led by medical Spanish faculty who routinely teach clinical communication and facilitate SP encounters, and who had previously delivered modules in this curriculum using the same format.

In preparation for each weekly session, students completed approximately 1 hour of directed self-study using assigned readings from a required medical Spanish textbook.^18^ Content included reproductive anatomy and physiology, clinical terminology, and relevant grammar structures to be applied during in-class activities. This preparatory work was intended to support active participation in task-based learning and SP encounters, although completion was not formally assessed.

At the beginning of the module, students completed a Spanish proficiency assessment and were assigned to either a beginner or intermediate/advanced track. Instructional sessions and embedded SP encounters were delivered separately for each track to support appropriate pacing and level-specific language development. Both tracks used the same materials, content, SP cases, and instructional time; they differed only in teaching pace and the amount of English used for support, with more English clarification in the beginner track and greater reliance on Spanish in the intermediate/advanced track. Within each group, students participated in small-group activities and were assigned rotating roles during clinical encounters to support engagement with different components of the medical interview.

Instruction was delivered by medical Spanish faculty with training in clinical communication and experience teaching Spanish in medical education settings.

The intervention was approved by the Institutional Review Board of the California University of Science and Medicine, under Protocol No. HS-2025-33, dated April 29, 2025.

### Equipment/Environment

Large-group sessions were conducted in a multimedia classroom equipped with multiple projection screens, ceiling microphones, and movable seating to facilitate interaction. Instruction was delivered primarily in Spanish to support an immersive language environment, with limited English clarification provided in presentation materials for the beginner group when necessary to support comprehension of clinically specific terminology. Students were seated in small table groups to promote interaction and prepare for subsequent task-based activities. Learners then remained at their tables for SP encounters, with each table functioning as a simulated outpatient visit.

### Implementation

The instructional design followed a progressive sequence to support clinical Spanish communication. Learners were introduced to reproductive health terminology during classroom sessions, applied these skills in small-group activities and SP encounters, and demonstrated integration of these skills during the OSCE.

Instructional materials included a facilitator guide (Appendix A), session-specific materials (Appendices B-D), and SP cases (Appendices E-G).

Each week consisted of a 60-minute instructor-led session followed by a 25-minute SP encounter. Sessions progressed from reproductive history-taking (eg, menstruation) to pregnancy and birth communication and finally to reproductive physical examination and assessment. SP encounters were conducted in small groups of 4 learners using cases aligned with weekly topics.

**Figure 1.**
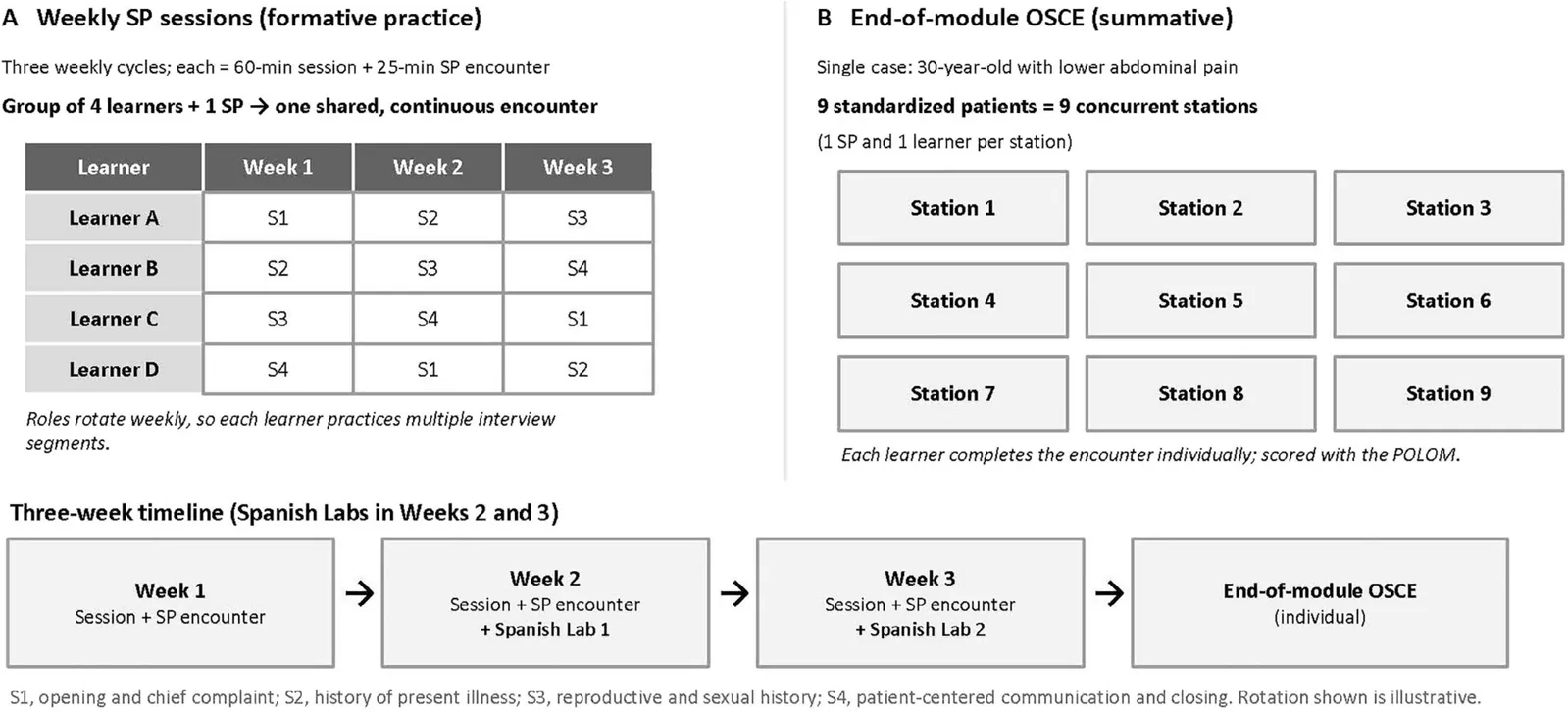
Three-week structure of the reproductive medical Spanish module. Each week included a 60-minute large-group lesson followed by a 25-minute small-group standardized patient (SP) encounter; learners worked in groups of 4 and rotated interview roles across the 3 weeks. Two 60-minute small-group Spanish labs were held in weeks 2 and 3. The module concluded with a 12-minute individual OSCE in which each learner completed the same single case, administered concurrently across 9 SP stations.

Learners worked in groups of 4 with a single SP and completed 1 encounter per session (3 encounters over the module). Rather than each learner conducting a separate full encounter, the group jointly conducted 1 continuous encounter, with each learner leading a distinct segment of the interview (opening and chief complaint, history of present illness, focused reproductive history, and patient-centered communication and closing). Assigned roles rotated across the 3 weekly sessions so that, over the module, each learner practiced multiple components of the encounter (Figure 1). Sessions were staffed by a faculty facilitator and an SP educator. Faculty observed each encounter, provided real-time guidance, and led a brief debrief on communication and language use. Each case specified the expected (critical) student actions for the encounter (see Appendices E-G), which guided the facilitator's observations and feedback. In contrast, the end-of-module OSCE was completed individually: each learner independently conducted the same single case, delivered concurrently across 9 SP stations (see Figure 1).

Students also participated in 2 small-group Spanish labs (Appendices H and I), held as separate sessions in weeks 2 and 3 (distinct from the weekly 60-minute lesson and 25-minute SP encounter) and aligned with the clinical skills curriculum. These sessions provided low-stakes practice through vocabulary review, case-based exercises, and structured interviews, reinforcing skills prior to SP encounters and the OSCE.

### Personnel

Each session required 1 faculty facilitator, 1 SP educator, and 1 SP per group of 4 learners. SPs were recruited from an existing institutional pool and selected for Spanish-language proficiency and prior experience in clinical simulation. Each SP was assigned to a specific case and was responsible for portraying the scenario consistently across learner groups and providing structured feedback following each encounter.

SP training was led by the institutional SP educator in collaboration with the course director. Training sessions included case review, role-play, and guidance on portrayal consistency, expected learner interactions, and feedback delivery. SPs were instructed to provide consistent responses, maintain role fidelity, and use a structured feedback checklist (Appendix J) to deliver brief feedback focused on clarity of communication and comprehensibility of Spanish.

The faculty facilitator supervised learner performance and provided feedback, whereas the SP educator ensured case fidelity and standardized portrayal across encounters.

Detailed SP recruitment criteria, training procedures, portrayal instructions, and feedback tools are provided in Appendix J.

### Debriefing

Following each SP encounter, we conducted a brief faculty-guided debriefing focused on clinical communication and Spanish language use. Debriefing followed a structured reflection format, including self-assessment, facilitator-guided discussion, and targeted feedback. Facilitators prompted learners to reflect on their performance, including clarity of expression, organization of the clinical interview, and use of appropriate reproductive health terminology. The discussion emphasized strategies for maintaining patient-centered communication in sensitive reproductive health contexts, including ensuring comfort, using inclusive language, and avoiding assumptions. Faculty also reinforced key language structures and addressed common errors observed during the encounters. SPs contributed brief feedback on comprehensibility and interpersonal communication. Debriefing prompts and facilitator guidance are provided in Appendix A, with corresponding model language, case-based examples, and session-specific teaching materials provided in Appendices B-D.

### Evaluation

SP encounters were used for guided practice and feedback, whereas the OSCE served as the primary performance-based assessment of learner outcomes. Learner performance was scored using the Physician Oral Language Observation Matrix (POLOM),^19^ an observational rating instrument adapted from the Student Oral Language Observation Matrix to assess medical oral language proficiency. The POLOM evaluates 6 domains—comprehension, fluency, vocabulary, pronunciation, grammar, and communication—each rated on a standardized ordinal scale; summed domain scores were converted to a percentage-based overall score (Appendix K: POLOM Rating Tool, Scoring Guide, and Rater Orientation). The POLOM was developed and validated using video-recorded medical student–SP encounters in US medical Spanish programs, where rater-based scores demonstrated convergent and criterion-related validity against established proficiency measures (the Interagency Language Roundtable–Health scale and the Clinician Cultural and Linguistic Assessment).^19,20^ Because that validation population (medical students assessed during Spanish-language SP encounters) and construct (medical Spanish oral proficiency) align directly with our learners and assessment format, the POLOM provides appropriate validity evidence for use in this context.

At the end of the module, students completed a mandatory OSCE assessing focused history-taking and patient-centered communication; physical examination and counseling were not assessed. The OSCE used a single SP case—a 30-year-old presenting with lower abdominal pain—aligned directly with the module's educational objectives. Each learner independently completed the same encounter, conducted entirely in Spanish, eliciting the chief complaint, clarifying symptom characteristics, and obtaining a relevant reproductive and menstrual history while demonstrating patient-centered communication. Each learner completed a single OSCE encounter (1 case) at 1 station. The same case was run concurrently at 9 SP stations so the cohort could be assessed in parallel; each station was staffed by 1 SP. The OSCE SP case is provided in Appendix L, and the door note and learner instructions in Appendix M. The OSCE case defines the expected (critical) student actions for the encounter (see Appendix L).

The OSCE grading team consisted of 5 raters: 2 full-time faculty members (1 clinician and the course director) and 3 additional raters with experience in clinical care, Spanish-language instruction, or health sciences education. All 5 raters were proficient in Spanish, consistent with the POLOM's requirement that raters be fluent in the target language. To promote scoring consistency, raters completed a standardized orientation prior to the OSCE, delivered as a live, synchronous virtual (videoconference) session lasting approximately 60 minutes. Training included review of the 6 POLOM domains and scoring conventions, discussion of scoring expectations, and group calibration in which raters independently scored illustrative sample encounters and reconciled their ratings to consensus. A rater orientation and calibration guide—including the scoring protocol and de-identified, scripted sample responses for practice—is provided in Appendix K to support replication. Each encounter was video-recorded using SimCapture, and the recordings were scored after the session by the 5-member faculty rater team. Raters independently scored the recorded encounters, applying the rubric consistently and distinguishing among levels of performance across domains.

## Results

A total of 131 second-year medical students participated in the module, and 128 completed the OSCE and were included in the analysis.

The mean OSCE score was 77.42% (*SD* = 2.28), with a median of 78% and a range of 68% to 92%. A total of 53.9% of learners achieved scores of 80% or higher, and 87.5% scored 70% or higher. These performance metrics are summarized in the Table.

**Table.**
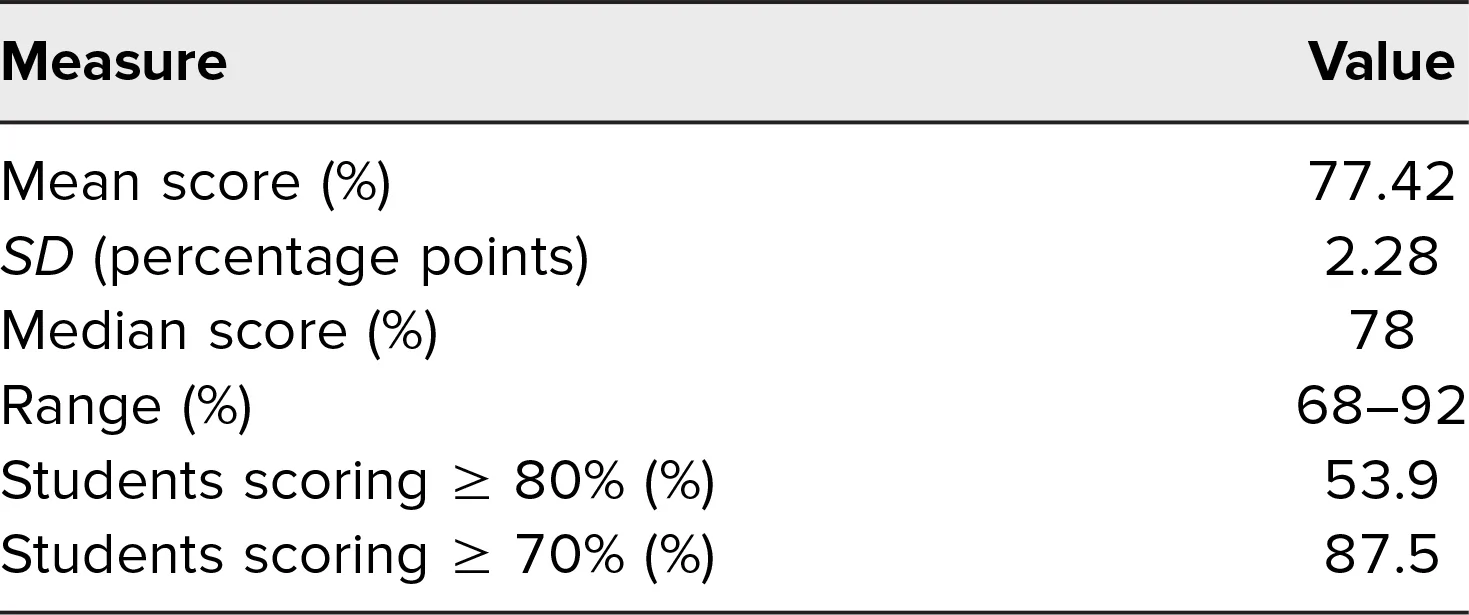
Summary of Participant Performance on the Reproductive System Spanish OSCE (*N* = 128)

**Figure 2.**
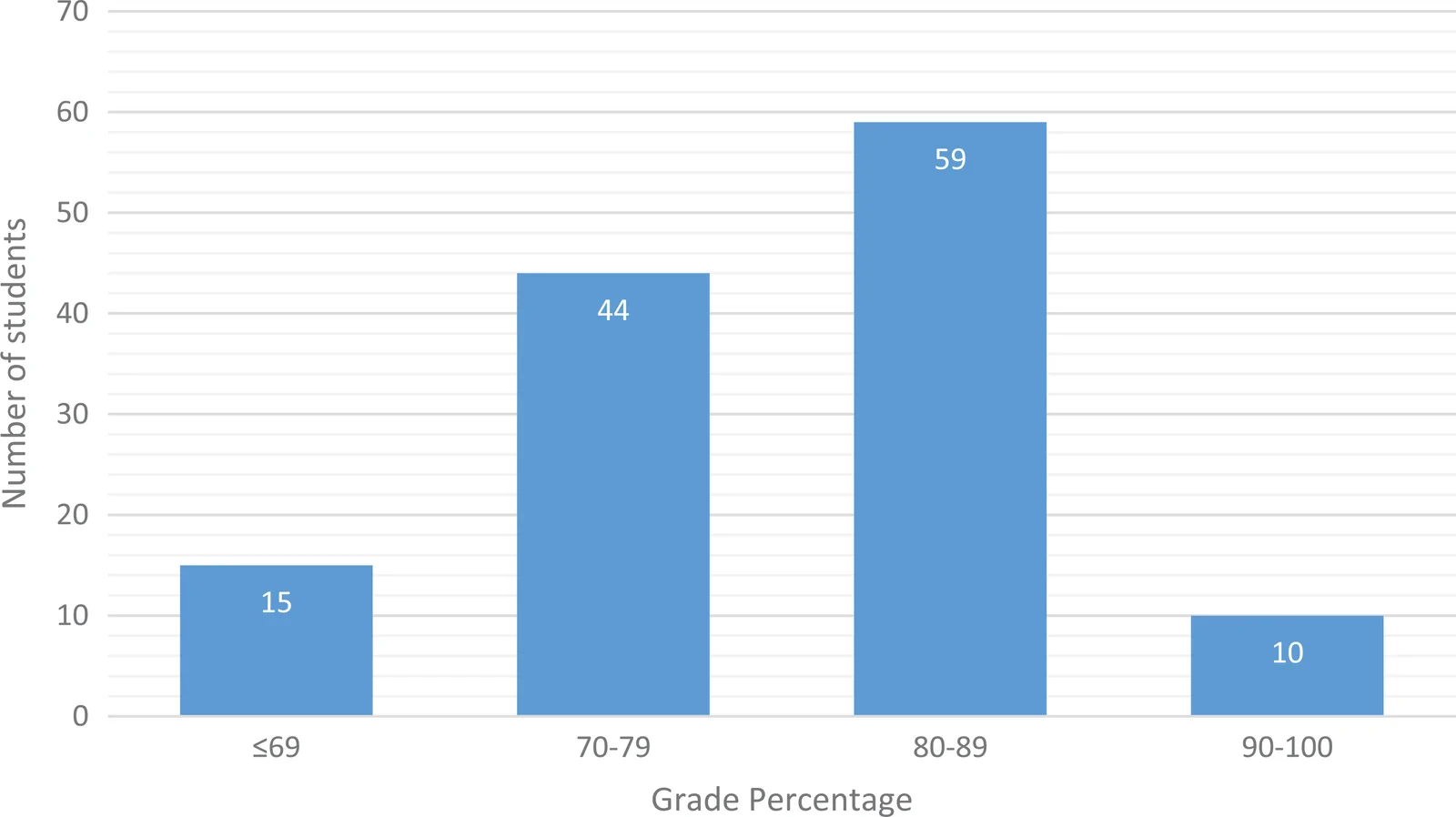
Distribution of Spanish-language OSCE performance scores among second-year medical students (*N* = 128).

Overall OSCE scores varied across learners, as shown by the distribution in Figure 2. At their station, each learner completed the core clinical communication tasks defined by the case—opening the encounter, eliciting the chief complaint, obtaining a focused reproductive health history, and using patient-centered communication strategies.

Evaluation data reflected both learner reaction (Kirkpatrick level 1) and observed performance (Kirkpatrick level 2). Learner reaction data were obtained through end-of-course evaluations, with 121 of 128 students (94.5%) completing the survey. Students reported high levels of satisfaction with the module structure, learning environment, and clinical relevance, with mean ratings ranging from 3.7 to 3.8 on a 4-point scale across items assessing organization, opportunities for practice, and perceived improvement in Spanish-language communication. A total of 76% of students strongly agreed and 21% agreed that the module improved their clinical Spanish communication, and 77% strongly agreed and 20% agreed that it strengthened their confidence and proficiency. Qualitative responses highlighted the value of SP encounters, structured practice opportunities, and clinical applicability of the content. When asked what could be improved, learners most commonly requested additional time for SP practice, and smaller group sizes to increase individual speaking opportunities. The end-of-module evaluation survey is presented in Appendix N.

## Discussion

This educational innovation addressed a gap in undergraduate medical education by providing structured training in reproductive health communication in Spanish within a systems-based curriculum. By integrating instructor-led sessions, guided practice, and SP encounters, the module enabled learners to apply reproductive health terminology and structured interview strategies.

OSCE performance suggests that learners were able to apply reproductive health communication skills in Spanish within a simulated clinical context. These findings extend prior work in medical Spanish education, which has largely focused on general clinical communication or other organ systems, by providing an applied model for integrating reproductive health content within a structured, experiential curriculum. Learner reaction data further indicated high levels of satisfaction and perceived improvement in Spanish-language communication and confidence, suggesting that the instructional design, including repeated SP encounters and structured practice, was well received and associated with learner engagement in sensitive clinical communication tasks.

Evidence that the module's objectives were met derives from criterion-referenced performance on the reproductive OSCE. On the POLOM, 87.5% of learners scored 70% or higher and 53.9% scored 80% or higher, indicating that the large majority demonstrated effective Spanish-language communication during a focused reproductive health encounter; learners completed the core tasks defined by the objectives—eliciting the chief complaint, obtaining a focused reproductive and menstrual history, and demonstrating patient-centered communication. The SP checklist (see Appendix J) was used to structure formative feedback during the weekly practice encounters rather than as a scored summative instrument; OSCE-based performance is therefore reported as the primary evidence of objective achievement.

Several features of our setting supported implementation and should be weighed by those wishing to replicate the module. It was embedded in an established, required longitudinal medical Spanish curriculum with proficiency-based tracking, Spanish-speaking faculty trained in clinical communication, an institutional SP pool with Spanish-proficient SPs, a dedicated SP educator, and timing aligned with the reproductive system block. Programs without these resources can still adapt the module: it can be offered as an elective or a shorter series, bilingual faculty or trained community members can serve as SPs, and the preparatory readings can be replaced with comparable materials that introduce reproductive health terminology in Spanish (see Appendix A). Key lessons learned were that a shared group encounter with rotating, defined roles allowed learners across proficiency levels to participate meaningfully; that proficiency-based tracks helped calibrate pacing and the amount of English support; and that learners valued repeated SP practice while requesting more individual speaking time and smaller groups—an adjustment planned for future iterations. Aligning the module with the concurrent reproductive block reinforced both language and clinical learning and is recommended where feasible.

This work has several limitations. The module was implemented at a single institution within a required medical Spanish curriculum, which may limit applicability to settings without similar curricular structures or faculty expertise. Evaluation relied on simulated encounters and did not assess communication in real clinical settings. The absence of a true preintervention assessment limits conclusions regarding change in performance attributable to the module. The relatively narrow score range may limit the detection of subtle differences in performance. Learner reaction data were based on self-reported evaluations and may not reflect objective measures of confidence or skill.

Future directions include expanding case content to include reproductive counseling, contraceptive decision-making, and communication surrounding early pregnancy loss, as well as incorporating additional assessment approaches such as multi-station OSCEs and longitudinal evaluation during clinical clerkships. Learner suggestions—particularly more speaking practice and more time with SPs—will inform future iterations of the module. Because the present module focused on the reproductive health of biological female patients, future modules could extend this approach to reproductive health more broadly, the reproductive health of biological males, and the reproductive health of transgender and gender-nonconforming individuals. Further work is needed to examine whether performance in simulated encounters translates to improved communication in clinical settings and to explore implementation across different institutional contexts.

## Appendices


Facilitator Guide.docxLesson 1 - Menstruation and Menstrual History.pptxLesson 2 - Pregnancy and Birth Communication.pptxLesson 3 - Reproductive Physical Exam.pptxSP Case Lesson 1.docxSP Case Lesson 2.docxSP Case Lesson 3.docxSpanish Lab 1 - Reproductive Communication.pptxSpanish Lab 2 - Reproductive History & Exam.pptxSP Recruitment.docxPOLOM Rating Tool Scoring Guide.docxOSCE SP Case.docxDoor Note and Learner Instructions.docxEnd-of-Module Evaluation Survey.docx
*All appendices are peer reviewed as integral parts of the Original Publication.*
