# Supplementary material for: Reproductive Health Communication in Spanish: A Standardized Patient-Based Module for Second-Year Medical Students
Source: MedEdPORTAL. 2026 Sep 8;22:11635. doi: 10.15766/mep_2374-8265.11635 (PMC13550226; doi:10.15766/mep_2374-8265.11635)
Supplement: Supplementary file 1 [file mep_2374-8265.11635-s001.zip › A. Facilitator Guide.docx]

**Appendix A.** Facilitator’s Guide

**Facilitator's Guide**

*These sessions are designed for preclerkship medical students with prior foundational training in Medical Spanish.*

*Lesson plans refer to large-group sessions; Spanish Labs refer to small-group facilitated sessions.*

**Three-Week Module Agenda**

The module spans three weeks. Each week includes approximately one hour of directed self-study (assigned readings from the course textbook), a 60-minute large-group lesson, and a 25-minute standardized patient (SP) encounter. Two 60-minute small-group Spanish Labs and a final individual OSCE complete the module.

**Week 1 — Medical Spanish for Reproductive Health I (menstruation and menstrual history):** ~1 hour directed self-study; 60-minute lesson; 25-minute SP encounter.

**Week 2 — Medical Spanish for Reproductive Health II (pregnancy and birth communication):** ~1 hour directed self-study; 60-minute lesson; 25-minute SP encounter.

**Week 3 — Medical Spanish for Reproductive Health III (reproductive examination and assessment):** ~1 hour directed self-study; 60-minute lesson; 25-minute SP encounter.

**Spanish Lab 1 and Spanish Lab 2:** 60 minutes each, small-group.

**Final assessment:** individual OSCE, 12 minutes per learner.

**Total instructional contact time:** approximately 6.25 hours (three 60-minute lessons, three 25-minute SP encounters, and two 60-minute Spanish Labs), plus approximately 3 hours of directed self-study and the final OSCE.

General Implementation Guidance

- **Recommended group size:** 4 learners per table.
- **Recommended personnel:** 1 faculty facilitator and 1 SP educator per session; 1 SP per table for SP activities.
- **Role rotation:** Rotate roles across sessions so each learner participates in different components of the interview over the 3-week module.
- **Feedback priorities:** Prioritize clarity, patient-centeredness, inclusivity, organization, and comprehensibility over grammatical perfection.
- **Timing:** If time is limited, preserve the SP activity and debrief; shorten large-group discussion first.
- **Scope:** These sessions emphasize communication skills in Spanish, not diagnostic mastery or performance of a physical exam.

Standardized Role Assignments for SP Activities

- **Primary interviewer:** Opens the encounter, confirms patient identity, establishes comfort/privacy, and elicits the chief complaint.
- **Historian:** Expands the history of present illness with focused follow-up questions.
- **Medical/family historian**: Obtains relevant past medical and family history.
- **Social historian:** Elicits social and sexual history using respectful, non-assumptive language.
- **Clinical reporter** (used when a fifth learner is present, or rotated across sessions): Summarizes key findings and likely concern to the group.
- **Physical-exam communicator** (Lesson 3 only): Explains the exam, requests consent, gives respectful instructions, checks comfort, and reports findings briefly.

Role Assignment Guidance: Facilitators should assign roles at the start of each SP activity and rotate them across sessions so each learner participates in different parts of the encounter during the module.

Debriefing Framework (applies to all sessions and labs)

Each session concludes with a brief structured debrief led by the facilitator. Debriefing should focus on three domains:

- **Communication effectiveness** (clarity, tone, organization)
- **Patient-centeredness** (comfort, inclusivity, non-assumptive language)
- **Language use** (appropriate vocabulary and comprehensibility)

Facilitators should:

- prioritize functional communication over grammatical accuracy
- reinforce effective phrasing through modeling
- correct only errors that interfere with meaning or patient comfort

**Lesson plan 1:** Medical Spanish for Reproductive Health I

**Target content:**

Reproductive anatomy, menstruation, menstrual cycle, common menstrual symptoms, inclusive and patient-centered language, cultural beliefs related to menstruation, and structured menstrual history-taking in Spanish.

**Duration:** 60 minutes.

**Objectives:**

1. Elicit a focused menstrual history in Spanish using patient-centered and inclusive language appropriate for a reproductive health encounter.
2. Respond to common cultural beliefs or concerns about menstruation using empathetic, nonjudgmental Spanish that supports patient comfort and disclosure.
3. Collaborate in a task-based clinical interaction to identify a likely menstrual concern and communicate an initial explanation and next steps in Spanish.

**Materials:**

1. PowerPoint Appendix B.

**Before the lesson:**

Prior to the session, students completed asynchronous self-study using the course textbook *Hablamos Vida: Medical Spanish for Student Doctors*.^[1]^ Assigned sections reviewed reproductive anatomy vocabulary, the menstrual cycle, common menstrual symptoms, and inclusive, patient-centered language related to menstruation and pregnancy. Use of this textbook reflects the local curricular context and is not required for other institutions implementing this module; facilitators may substitute any comparable preparatory materials that introduce foundational reproductive health terminology in Spanish.

**Lesson Plan:**

**1. Warm-up & Activation (10 minutes)**

**Slides:**

- “¿Qué sabe usted del ciclo menstrual?”
- Terms for menstruation (Spain / Latin America / Mexico)

**Implementation:**
Students work in pairs or small groups to discuss everyday terms patients use for menstruation and cultural beliefs they have heard. The facilitator invites examples from different regions and briefly normalizes variation in language use. The goal is to move students from everyday Spanish into clinical relevance without correction or lecturing.

**2. Cultural Beliefs & Empathic Responses (5 minutes)**

**Slides:**

- Menstrual myths
- Empathic language guide

**Implementation:**
As a large group, review common beliefs shown on the slide. Ask students how they might respond respectfully rather than correcting the belief directly. The facilitator models short empathic phrases in Spanish and asks students to repeat or paraphrase them aloud.

**3. Vocabulary & Clinical Questions (10 minutes)**

**Slides:**

- Key vocabulary
- Menstrual history questions

**Implementation:**
This is a **guided review**, not memorization. The facilitator asks students to read questions aloud, identify what each question assesses (timing, flow, pain), and practice asking one question to a peer. Emphasis is on clarity, tone, and patient comfort rather than grammatical precision.

**4. Case-Based Communication Activity (5 minutes)**

**Slides:**

- Case-based activity (Cases 1–2)
- Inclusive language examples

**Implementation:**
Students briefly work in groups to identify a likely diagnosis and next steps for one case. The facilitator calls on different groups to share ideas and asks others whether they agree before showing the model examples on the slides. This keeps the activity collaborative and avoids turning it into a lecture.

**Facilitator Notes / Model Output**

- Acceptable student responses should identify a likely menstrual concern and justify it using symptom details from the case.
- Ask groups to share one Spanish question they would ask next and one patient-centered response.
- Model follow-up should emphasize symptom clarification, reassurance, and next steps rather than definitive diagnosis.
- Common correction points: overly direct questioning, lack of empathy, and failure to normalize variation in menstrual language.

Example model language: “¿Cuándo empezó su último período?” / “Entiendo que esto puede ser incómodo de hablar, pero gracias por contármelo.”

**5. Task-Based Standardized Patient Activity (25 minutes)**

**Slides:**

- Task-based activity
- Roles per table
- Possible diagnoses

**Implementation:**
Students work in groups of four with one standardized patient per table. Roles are assigned by the instructor to ensure rotation across sessions:

- Primary interviewer
- Medical/family historian
- Social historian
- Clinical reporter

All students collaborate to reach a diagnosis. Faculty and SP educator circulate, offering brief, targeted feedback focused on communication, inclusivity, and use of reviewed language.

**Facilitator Notes / Model Outpu**t:

- Faculty should allow the interaction to continue unless communication breaks down or students shift fully into English.
- Feedback should focus on inclusivity, confidentiality, tone, and question sequencing rather than isolated grammar errors.

**6. Debrief and Reflection (5 minutes)**

**Implementation:**
Bring the group back together and ask:

- What language felt most useful today?
- What felt hardest to ask?
- What would you say differently next time?

Briefly connect today’s work to upcoming reproductive sessions and the final OSCE, reinforcing that this session focused on **using language in context**, not mastery.

Key teaching points to reinforce

•	Menstrual terminology varies across regions and patients.

•	Empathic responses improve comfort and disclosure.

•	Clear, nonjudgmental questioning is more important than perfect grammar.

**Lesson plan 2:** Medical Spanish for Reproductive Health II

**Target content:**

Pregnancy and birth–related vocabulary, common cultural beliefs and myths about pregnancy, patient-centered and inclusive language, selected pregnancy risk factors, and structured pregnancy history-taking and counseling in Spanish.

**Duration:** 60 minutes.

**Objectives:**

1. Apply basic Spanish vocabulary for pregnancy, prenatal care, and birth (e.g., trimesters, due date, prenatal checkups).
2. Address common pregnancy myths and reformulate them into simple clinical language in Spanish.
3. Participate in a supported, task-based SP encounter focused on pregnancy history-taking/counseling and propose a likely diagnosis as a group.

**Materials:**

1. PowerPoint Appendix C.

**Before the lesson:**

Prior to the session, students completed asynchronous self-study using the course textbook *Hablamos Vida: Medical Spanish for Student Doctors*.^[2]^ Assigned sections focused on pregnancy and birth–related vocabulary, common pregnancy myths and beliefs, and patient-centered, inclusive language for discussing pregnancy, labor, and delivery in Spanish. Use of this textbook reflects the local curricular context and is not required for other institutions implementing this module; facilitators may substitute any comparable preparatory materials that introduce foundational pregnancy and birth terminology in Spanish.

**Lesson Plan:**

**1. Warm-up & Activation (10 minutes)**

**Slides:**
Pregnancy myths and common statements

**Implementation:**
Students work in pairs to discuss the statements presented on the slide and decide whether they believe each one is true or false. They are asked to briefly explain their reasoning and, when possible, restate the idea using more clinical or neutral language in Spanish. The facilitator then brings the class together to review the statements, inviting different pairs to share their perspectives. This activity activates prior knowledge and helps students transition from everyday beliefs to clinically relevant language without correcting or lecturing.

**2. Vocabulary & Clinical Framing (10 minutes)**

**Slides:**
Pregnancy, birth, and labor vocabulary

**Implementation:**
The facilitator introduces the vocabulary slide as a review and application activity rather than memorization. Students work in pairs to create a short, simple sentence in Spanish using several of the listed terms. A few volunteers share their sentences aloud. The facilitator reinforces clarity and patient-friendly phrasing, emphasizing that the goal is comfort using the language in context.

**3. Risk Factors & Guided Discussion (10 minutes)**

**Slides:**
Pregnancy-related risk factors
Why it matters / key questions / model phrase format

**Implementation:**
The facilitator signals a shift to discussing selected pregnancy-related risk factors and briefly introduces each condition using the slide structure. After each condition, students turn to a partner and choose one question they would realistically ask a patient during a pregnancy visit. Several students share their questions aloud. The focus remains on how to ask about risk clearly and respectfully, rather than on medical detail.

**Facilitator Notes / Model Output**

- Students should formulate simple, respectful questions that screen for pregnancy-related risk factors without sounding accusatory.
- Model language should emphasize clarity, tone, and patient-friendly wording.
- Common correction points: vague wording, overly technical language, and abrupt transitions into sensitive topics.

Example model language: “¿Ha tenido atención prenatal?” / “Quiero hacerle unas preguntas para entender mejor su embarazo y apoyarle de la mejor manera.”

**4. Task-Based Standardized Patient Activity (25 minutes)**

**Slides:**
Task-based activity instructions
Roles per table
Possible diagnoses

**Implementation:**
Students work in groups of four with one standardized patient at each table. The instructor assigns roles to ensure balanced participation across sessions: primary interviewer, medical/family historian, social historian, and clinical reporter. Students collaborate to conduct the encounter and arrive at a likely diagnosis together. Faculty and SP educator circulate to support communication, inclusivity, and use of reviewed language, offering brief feedback without interrupting the flow of the interaction.

Faculty should listen for respectful pregnancy history-taking, inclusive language, logical question sequencing, and avoidance of assumptions.

**5. Debrief and Reflection (5 minutes)**

**Slides:**
Diagnosis example and patient-centered plan

**Implementation:**
The facilitator brings the group back together and briefly contextualizes why pregnancy communication matters, then projects the diagnosis slide. One example of patient-centered diagnostic explanation and plan language is modeled aloud. The session closes by reinforcing that the focus was on applying language in context and preparing for continued practice and the final OSCE.

**Key teaching points to reinforce**

- Pregnancy myths should be addressed respectfully rather than dismissed.
- Students should use plain, patient-centered Spanish when discussing risk and next steps.

**Lesson plan 3:** Medical Spanish for Reproductive Health III

**Important implementation note:**

This session focuses on verbal communication used during a reproductive physical exam (consent, explanation, positioning, comfort checks, and next-step language). Learners do not perform an actual physical exam.

**Target content:**

Immediate-future clinical planning language using *ir + a + infinitivo*, respectful formal instruction-giving for patient guidance, and high-yield basic reproductive physical exam phrases (consent, positioning, step-by-step instructions, discomfort check-ins, brief reporting of findings), practiced through a supported task-based standardized patient encounter

**Duration:** 60 minutes.

**Objectives:**

1. Express an immediate clinical plan aloud in Spanish using *ir + a + infinitivo*
2. Give clear and respectful patient instructions using formal commands, recognize and use core phrases for a brief reproductive physical exam, and apply these language functions during a supported standardized patient encounter

**Materials:**

1. PowerPoint Appendix D.

**Before the lesson:**

Prior to the session, students completed asynchronous self-study using the course textbook *Hablamos Vida: Medical Spanish for Student Doctors*.^[3]^ Assigned sections focused on introductory physical-exam communication in Spanish within the reproductive block, including patient-centered permission language, immediate-plan statements used during exams, and common instruction-giving phrases. Use of this textbook reflects the local curricular context and is not required for other institutions implementing this module; facilitators may substitute any comparable preparatory materials that introduce foundational reproductive exam communication in Spanish.

**Lesson Plan:**

**1. Warm-up & Activation (5 minutes)**
Open with the quick recall question on the slide (the “who remembers” prompt) to activate what students already recognize. Keep it low-stakes and fast—one or two student examples is enough to launch the session.

**2.** “**Plan language” in context (5 minutes)**
Use the immediate-plan examples as clinical talk, not as a grammar lecture. Have pairs choose two of the clinical infinitives on the slide (for example, *examinar*, *explicar*, *tomar la presión*) and produce a short “what I’m going to do next” sequence they could actually say during an encounter (two lines per pair). Invite a few pairs to say theirs out loud, then briefly model one polished version.

**3. Patient instructions in context (10 minutes)**
Introduce formal instructions as “the language of clear, respectful guidance.” Students work in pairs to transform a neutral instruction into something they would actually say during an exam, using the slide models and a respectful tone (adding *por favor*, *voy a pedirle que…*, etc.). You circulate and offer short, targeted coaching on clarity and patient comfort.

**4. Physical exam phrases (10 minutes)**
Move into the reproductive physical exam phrase bank and run it as a mini rehearsal. Pairs practice a short exam script: obtain consent, give one changing/positioning instruction, check comfort, and close with a brief “you can get dressed” line. Emphasize pacing, permission, and reassurance.

**5. Task-based standardized patient activity (25 minutes)**
Set expectations: one standardized patient per table, four students per table, and roles assigned by the instructor to ensure rotation. Include the physical-exam role explicitly: the **physical examiner** explains the exam, requests consent, gives clear respectful instructions during the exam, and reports key findings briefly to the team. Students support each other to complete the clinical task, and faculty/SP educator circulate to keep the encounter moving and give brief feedback focused on communication, inclusivity, and use of the reviewed language.

If a learner attempts to go beyond verbalized exam communication, the facilitator or SP should redirect the interaction back to explanation, consent, and instruction-giving language.

**Facilitator Notes / Model Output**

Students should focus on verbalizing consent, positioning instructions, comfort checks, and next-step language clearly and respectfully. Facilitators should reinforce that the goal is communication during exam preparation, not physical exam performance.

**6. Debrief and Reflection (5 minutes)**
Bring everyone back together and ask two quick debrief questions: what phrase felt most useful today, and what felt hardest to say. Close by reinforcing that today’s goal was functional exam communication (consent, instructions, comfort checks, and next-step language) in preparation for the upcoming reproductive OSCE.

**Key teaching points to reinforce**

- Consent and comfort checks should be explicit.
- Instructions should be clear, respectful, and paced slowly.
- Exam language should support patient autonomy and understanding.

**Spanish Lab 1:** Medical Spanish for Reproductive Health I (Small Group Lab)

**Target content:**

Barriers to discussing reproductive health, reproductive system vocabulary, menstrual and sexual health–related symptoms, inclusive and patient-centered language, confidentiality, and formulation of respectful clinical questions in Spanish across reproductive contexts.

**Duration:** 60 minutes.

**Objectives:**

1. Identify common linguistic, cultural, and structural barriers to discussing sexual and reproductive health with Spanish-speaking patients.
2. Formulate clear, inclusive, and patient-centered reproductive health questions in Spanish across multiple clinical scenarios.
3. Use trust-building and confidentiality-oriented language to support disclosure in sensitive reproductive health encounters.

**Materials:**

1. PowerPoint Appendix H.

**Lesson Plan:**

**1. Warm-up & Activation: Barriers to Reproductive Health Communication (5 minutes)**

**Slide:**
¿Qué barreras existen al hablar de salud sexual con pacientes hispanohablantes?

**Implementation:**
Students work briefly in pairs or small groups to identify three barriers to discussing sexual or reproductive health in Spanish-speaking patients (e.g., stigma, fear of disclosure, generational beliefs, confidentiality concerns, language limitations). The facilitator then invites groups to share examples aloud and briefly synthesizes themes. Emphasis is placed on normalizing discomfort and framing barriers as contextual rather than individual failures.

**2. Case-Based Question Formulation: Reproductive System Scenarios (15 minutes)**

**Slides:**
Two clinical cases (A–B).

**Implementation:**
Students work in pairs or small groups. For **each assigned case**, students complete three tasks:

1. Identify a likely diagnosis or concern.
2. Write three clinical questions in Spanish that would help clarify the diagnosis.
3. Write one trust-building or reassurance statement in Spanish appropriate to the case.

The facilitator circulates actively, offering real-time feedback focused on tone, inclusivity, and clarity rather than grammatical perfection. After each case, the facilitator briefly reviews model questions and trust-building language shown on the slides, highlighting why certain phrasing supports patient comfort and disclosure.

**Facilitator Notes / Model Output**

For each case, acceptable responses should include:

- a plausible concern or differential,
- at least 3 clear follow-up questions in Spanish, and
- 1 trust-building statement that supports disclosure.

Facilitators should model how confidentiality, empathy, and non-assumptive wording improve the quality of reproductive health questioning.

**3. Guided Discussion: Inclusive Language & Confidentiality (10 minutes)**

**Slides:**
Case-specific sample questions and trust-building phrases.

**Implementation:**
As a full group, review the example questions and phrases provided for each case. The facilitator asks students:

- What makes this question feel safer or more inclusive?
- How does confidentiality language change the interaction?
- How might wording differ for adolescents, adults, or LGBTQ+ patients?

Students are encouraged to paraphrase or adapt phrases aloud. The facilitator models inclusive language explicitly and reinforces that these phrases are transferable across clinical contexts.

**4. Structured Language Framework Practice (ABCDE / PIES) (10 minutes)**

**Slides:**
ABCDE del lenguaje clínico
PIES model (Preparar – Informar – Educar – Sostener)

**Implementation:**
The facilitator introduces or reviews the ABCDE and PIES frameworks as practical tools for structuring sensitive reproductive health conversations. Students practice applying the framework to one of the prior cases by orally sequencing how they would:

- Prepare the patient
- Provide information
- Offer education
- Provide support

This segment emphasizes organization of language and emotional safety rather than diagnostic accuracy.

This framework is used to organize sensitive communication, not to force students into a rigid script.

**5. Advanced Case: Communication Activity with Psychosocial Context (15 minutes)**

**Slides:**
Extended clinical case (29-year-old patient with dyspareunia/vaginismus features).

**Implementation:**
Students work in small groups to analyze the case, identify a likely diagnosis, and propose:

- Appropriate reproductive and psychosocial follow-up questions in Spanish
- One patient-centered explanation acknowledging physical and emotional contributors

The facilitator leads a brief whole-group debrief, guiding students toward recognizing the role of stress, muscle tension, and noninfectious etiologies while modeling nonjudgmental explanatory language in Spanish.

**Facilitator Notes / Model Output**

Students should recognize that pain with penetration may involve physical and psychosocial contributors and should avoid framing the concern as purely psychological or purely infectious. Model language should acknowledge discomfort, validate the patient’s experience, and invite further discussion without judgment.

**6. Debrief and Reflection (5 minutes)**

**Implementation:**
The facilitator closes by asking:

- What language felt hardest to use today?
- What phrasing felt most supportive or natural?
- How did your approach change when confidentiality or stigma was involved?

The session concludes by reinforcing that the goal of the lab is **functional, patient-centered communication**, not mastery, and by briefly connecting this work to upcoming reproductive health sessions and future OSCE expectations.

**Key teaching points to reinforce**

- Confidentiality language can improve disclosure.
- Inclusive wording supports trust in sensitive encounters.
- The goal is organized, supportive questioning rather than perfect grammar.

**Spanish Lab 2:** Medical Spanish for Reproductive Health II (Small Group Lab)

**Target content:**

Opening a reproductive health clinical encounter in Spanish; eliciting and developing the chief complaint; applying the SOCRATES framework to reproductive pain; asking case-specific reproductive questions; obtaining relevant family and social history; and using appropriate, patient-centered language to guide a reproductive physical exam.

**Duration:** 60 minutes.

**Objectives:**

1. Open a reproductive health clinical encounter in Spanish using inclusive, patient-centered, and confidentiality-focused language.
2. Elicit and expand the chief complaint using appropriate follow-up questions and the SOCRATES framework in reproductive contexts.
3. Formulate relevant case-specific, family history, and social history questions in Spanish to support reproductive clinical reasoning.
4. Use clear, respectful Spanish to guide a patient through a reproductive physical exam while maintaining comfort, consent, and professionalism.

**Materials:**

1. PowerPoint Appendix I.

**Lesson Plan:**

**1. Warm-up & Activation: Cultural and Linguistic Assumptions (5 minutes)**

**Slides:**
Verdadero o Falso – Sistema Reproductivo

**Implementation:**
Students work briefly in pairs or small groups to decide whether each statement is true or false (e.g., avoidance of anatomical terms, use of diminutives, regional variation in menstrual terminology, expectations around partner involvement). The facilitator reviews responses as a group, emphasizing that these statements reflect **common patterns**, not universal rules. The goal is to sensitize students to variation in patient language and expectations before practicing encounter skills.

**2. Opening the Clinical Encounter (10 minutes)**

**Slides:**
Abrir el encuentro clínico

**Implementation:**
The facilitator models a complete opening of a reproductive health encounter, including name, role, pronouns, patient comfort, confidentiality, and agenda setting. Students then practice the opening in pairs, alternating roles as clinician and patient. The facilitator circulates and provides targeted feedback on tone, clarity, pacing, and inclusivity rather than grammatical accuracy.

**Facilitator Notes / Model Output**

A strong opening should include role introduction, patient name confirmation, privacy/confidentiality language, a comfort check, and an agenda-setting question. Facilitators should prioritize warmth, clarity, and pacing over grammatical precision.

**3. Eliciting the Chief Complaint (5 minutes)**

**Slides:**
Elicitar el motivo de la consulta

**Implementation:**
Students practice open-ended questions to elicit and expand the chief complaint, followed by brief empathic statements. The facilitator pauses the group intermittently to highlight effective phrasing that encourages disclosure and avoids minimizing patient concerns. Emphasis is placed on listening and follow-up rather than rapid questioning.

**4. Pain Assessment Using SOCRATES (15 minutes)**

**Slides:**
SOCRATES – Encuentro clínico reproductivo

**Implementation:**
Working in pairs, students apply the SOCRATES framework to a reproductive pain scenario provided on the slide. One student asks questions while the other responds, then roles switch. The facilitator guides the group through each SOCRATES component, reinforcing how structured pain assessment supports clinical reasoning while maintaining patient comfort in sensitive contexts.

**Facilitator Notes / Model Output**

Students should use SOCRATES to structure questioning, but interactions should remain conversational rather than checklist-like. Facilitators should reinforce smooth transitions, empathy, and clarity when asking about pain in sensitive reproductive contexts.

**5. Case-Specific, Family, and Social History Questions (15 minutes)**

**Slides:**
Preguntas de caso específicas
Historial familiar
Historial social y hábitos

**Implementation:**
Students work in small groups to select appropriate follow-up questions across three domains: reproductive symptoms, family history, and social history. The facilitator prompts students to explain *why* a question is relevant and models how to transition smoothly between sections of the history. Attention is given to respectful inquiry around sexual practices, substance use, and psychosocial context.

Model transitions should include brief permission language before sexual, social, or psychosocial questions.

**6. Guided Physical Exam Language (5 minutes)**

**Slides:**
Examen físico – lenguaje guiado

**Implementation:**
The facilitator reviews and models step-by-step language used to guide a reproductive physical exam, emphasizing consent, clear explanations, and patient autonomy. Students practice saying key phrases aloud in pairs. The goal is familiarity with appropriate language, not performance of the exam itself.

The goal is guided verbalization of exam language only; learners do not perform or simulate exam maneuvers.

**7. Debrief and Reflection (5 minutes)**

**Implementation:**
Bring the group back together and ask:

- What part of the encounter felt most challenging to say in Spanish?
- Which phrases helped maintain patient comfort?
- How did structured tools like SOCRATES support your questioning?

The facilitator closes by reinforcing that this lab focuses on **functional, patient-centered clinical communication**, and connects the skills practiced to future reproductive labs and OSCE expectations.

**Key teaching points to reinforce**

- A strong opening sets the tone for the entire encounter.
- Permission language helps with sensitive transitions.
- Structured tools such as SOCRATES support clarity without replacing empathy.

**Common Facilitation Challenges**

- If students become too diagnosis-focused, redirect them to communication goals and question quality.
- If students rely heavily on English, prompt them to restate the question or phrase in simple Spanish.
- If a group is quiet, ask one learner to model a single opening line or follow-up question to restart participation.
- If wording becomes judgmental or assumptive, pause briefly and model a more patient-centered alternative.
- If one learner dominates, reassign the next question to a quieter role-holder.

1. Lopez-Vera A. Hablamos Vida: Medical Spanish for Student Doctors. 1st ed. Kendall/Hunt Publishing Company; 2025
2. Lopez-Vera A. Hablamos Vida: Medical Spanish for Student Doctors. 1st ed. Kendall/Hunt Publishing Company; 2025
3. Lopez-Vera A. Hablamos Vida: Medical Spanish for Student Doctors. 1st ed. Kendall/Hunt Publishing Company; 2025
