# Supplementary material for: Reproductive Health Communication in Spanish: A Standardized Patient-Based Module for Second-Year Medical Students
Source: MedEdPORTAL. 2026 Sep 8;22:11635. doi: 10.15766/mep_2374-8265.11635 (PMC13550226; doi:10.15766/mep_2374-8265.11635)
Supplement: Supplementary file 1 [file mep_2374-8265.11635-s001.zip › E. SP Case Lesson 1.docx]

**Appendix E.** Standardized Patient Case: Lesson 1 (Menstrual Concerns)

**Contexto clínico para facilitadores**
Referencia basada en evidencia para facilitadores y pacientes estandarizados. La tarea del estudiante es la comunicación en español; este contenido clínico se proporciona para la fidelidad del caso y no se evalúa.

**Diagnóstico de trabajo:** Trastorno disfórico premenstrual (TDPM); síndrome premenstrual (SPM) grave.

**Motivo de consulta:** Síntomas emocionales y físicos recurrentes en la fase lútea (los días previos a la menstruación) que se resuelven poco después del inicio de la menstruación.

**Diagnósticos diferenciales principales:** TDPM/SPM; exacerbación premenstrual de un trastorno del estado de ánimo o de ansiedad subyacente; disfunción tiroidea.

**Anamnesis basada en evidencia:** Confirmar el carácter cíclico (inicio en la fase lútea con resolución en los primeros días de la menstruación); impacto funcional/laboral; síntomas del estado de ánimo y de ansiedad; síntomas tiroideos; medicamentos y consumo de sustancias; antecedentes psiquiátricos.

**Exploración física:** No se requiere una exploración física específica; el diagnóstico es clínico y se basa en el registro prospectivo de los síntomas (signos vitales según corresponda).

**Evaluación/estudio inicial:** El registro diario y prospectivo de los síntomas durante al menos dos ciclos menstruales es el estándar diagnóstico; aplicar los criterios del DSM-5 para el TDPM; considerar TSH y tamizaje de depresión/ansiedad para descartar otras causas.

**Referencia:** Hofmeister S, Bodden S. Premenstrual Syndrome and Premenstrual Dysphoric Disorder. Am Fam Physician. 2016;94(3):236-240.

**Nombre del caso:** Caso de paciente estandarizado: evaluación reproductiva con síntomas emocionales y físicos severos previos a la menstruación**.**

**Tipo de encuentro:** Encuentro formativo con paciente estandarizado

**Escenario:** Ambulatorio

Formato: Grupo pequeño (4 estudiantes : 1 paciente estandarizado)

**Duración:** 25 minutos

**Tarea del estudiante:** Historia clínica reproductiva enfocada y comunicación centrada en el paciente en español

**Examen físico**: No se realiza; solo se verbaliza si corresponde

**Consejería:** No requerida

**Idioma requerido:** Solo español

**Evaluación:** Formativa (no calificada)

**Notas de facilitación específicas del caso**

Este caso está diseñado para la interacción en grupos pequeños (4 estudiantes por paciente estandarizado), con roles asignados según se describe en el Apéndice A.

Los facilitadores deben permitir que la interacción se desarrolle sin interrupciones, a menos que la comunicación se deteriore.

Enfoque de observación:

•	identificación del patrón cíclico de los síntomas

•	uso de lenguaje empático

•	exploración del impacto emocional y funcional

Este caso enfatiza la comunicación y el reconocimiento de patrones más que la precisión diagnóstica.

**Actividad Educacional Formativa**

**Métodos**

Los Pacientes Estandarizados (PE) recibieron previamente un guion detallado con el perfil del paciente, así como información sobre las características personales y el historial médico relevante, con el fin de familiarizarse adecuadamente con el caso antes de la simulación.

La actividad se llevó a cabo en el salón de clase. El encuentro clínico se desarrolló en mesas de trabajo, con un promedio de cuatro estudiantes por mesa y un Paciente Estandarizado, durante un período aproximado de 25 minutos.

Los materiales de capacitación incluyeron el guión clínico detallado del caso.

| ESCENARIO: Ambulatorio, hospitalizado, urgencias, hogar, residencia de ancianos, rehabilitación, grupo, etc. | Ambulatorio |
| --- | --- |
| PERFIL DEL PACIENTE: Información sobre el “paciente” que ayuda a seleccionar un SP y ayuda al aprendiz a comprenderlos como persona. El SP conocerá más información sobre el paciente de la que el aprendiz preguntará, pero permite que el SP represente una personalidad de paciente completamente desarrollada. Si ninguno de los elementos a continuación es particular para el caso, escriba “se pueden usar todos”. | |
| Nombre del paciente | Daniela Márquez |
| Pronombre | Ella |
| Rango de edad | 28 años |
| Religión/ antecedentes espirituales | Católica, no practicante |
| Sexo (masculino, femenino, intersexual, transgénero…) | Mujer cisgénero |
| Orientación sexual (por ejemplo, heterosexual, lesbiana, gay, bisexual, pansexual, queer, asexual) | Heterosexual |
| Expresión de género (por ejemplo, hombre, mujer, género no binario) | Mujer |
| Raza y/o etnia | Hispana/Latinx |
| Descripción física (por ejemplo, IMC, rango de altura) | No especificado; todos pueden usarse |
| Limitaciones físicas | Ninguna |
| Apariencia del paciente (por ejemplo, desaliñado, bata de hospital, informal de negocios) | Informal; Buen contacto visual, puede suspirar o emocionarse al hablar de sus síntomas |
| Simulación + ubicación (por ejemplo, ninguna, moretones, cicatrices, piercings, tatuajes) | Ninguna |
| Afecto (por ejemplo, agradable, cooperativo) | Cooperativa, Afecto triste y frustrado |
| El paciente debe: | Responder de manera abierta pero no ofrecer información no solicitada  Mostrar afecto triste y frustrado, especialmente al describir síntomas emocionales  Suspirar o hacer pausas al hablar de impacto en su vida diaria  Responder con más detalle cuando el estudiante usa lenguaje empático  Responder de forma breve si las preguntas son directas o poco empáticas  Si el estudiante usa lenguaje poco claro:  Pedir aclaración (“¿Puede repetir la pregunta?”)  Si el estudiante cambia a inglés:  Responder en español y mantener el encuentro en español |
| Grupo familiar (por ejemplo, quiénes son familiares, con quién viven) | Vive sola |
| Educación | Escuela secundaria completada; trabaja como diseñadora gráfica |
| Nivel de alfabetización en salud | Moderado |
| Empleo, si lo hay: presente y pasado, señalando cualquier estrés actual | Diseñadora gráfica |
| Hogar/sin hogar - tipo de vivienda, número de pisos, propiedad o alquilada | Vive sola |
| Situación financiera - cualquier estrés actual | Recursos limitados |
| Estado del seguro (por ejemplo, no asegurado/infrasegurado/asegurado, público/privado, HMO/PPO) | Sin seguro médico |
| Hábitos (es decir, dieta, ejercicio, cafeína, fumar, alcohol, drogas) | No fuma ni usa drogas recreativas; consumo ocasional de alcohol (1-2 veces al mes en reuniones) |
| Actividades (es decir, pasatiempos, deportes, clubes, amigos) | Le gusta leer y escuchar música |
| Día típico - cuál es la rutina diaria habitual | Trabaja como diseñadora gráfica, dividiendo su tiempo entre trabajo presencial y virtual según el día. |

| CASE INFORMATION | |
| --- | --- |
| Queja principal: Lo que el paciente dirá cuando sea recibido por el estudiante. La razón principal del paciente para buscar atención médica, a menudo expresada en sus propias palabras | “Vengo porque antes de que me baje la regla, me siento fatal. Me afecta mucho.” |
| Reglas de divulgación de información | Información inicial (sin que el estudiante pregunte):  - Síntomas generales previos a la menstruación  Solo si el estudiante pregunta específicamente:  Detalles del ciclo menstrual (duración, regularidad)  - Impacto en el trabajo  - Historial familiar de salud mental  - Actividad sexual y uso de anticonceptivos  Si el estudiante no pregunta:  - No ofrecer detalles sobre historial sexual ni familiar |
| Preocupaciones adicionales: Otras preocupaciones que el paciente tenga hoy (es decir, síntomas, solicitudes, expectativas, etc.) que se incluirán en la agenda establecida | “Cada mes, unos días antes de que me baje, me pongo súper irritable. Lloro por todo, me siento ansiosa, no duermo bien, y me duele el cuerpo. Y después, cuando ya me baja, se me pasa todo. Ya no sé si es normal.” |
| HISTORIA DE LA ENFERMEDAD ACTUAL: Aunque parte de la historia de la enfermedad actual se dará en el relato de los síntomas del paciente, los aprendices ampliarán la historia durante la sección de preguntas directas. A continuación, describa la historia detallada, generalmente sobre la queja principal, que el estudiante debe desarrollar para hacer una evaluación útil del problema: | |
| Inicio (cuándo; gradual o repentino) | Desde hace aproximadamente 8 meses, pero ha empeorado en los últimos 3 ciclos |
| Contexto (qué estaba pasando o dónde estaba el paciente cuando se notaron los síntomas por primera vez) | Inicio gradual, sin desencadenante claro |
| Duración (cuánto tiempo) | Los síntomas aparecen entre 7 y 10 días antes de la menstruación y desaparecen cuando comienza el sangrado |
| Relaciones de tiempo (frecuencia, constante o intermitente) | De manera constante durante aproximadamente 8 meses |
| Ubicación | No aplica |
| Radiación | Ninguna |
| Calidad | Moderada a severa. “A veces no puedo funcionar bien en el trabajo.” |
| Intensidad | No aplica |
| Empeorado por qué | El estrés del trabajo y dormir mal lo empeoran todo. |
| Aliviado por qué | Nada ha funcionado bien. A veces el ejercicio ayuda un poco. |
| Asociado con qué | - Llanto fácil - Cambios de humor repentinos - Insomnio - Irritabilidad intensa - Sensación de culpa después de discutir - Calambres leves - Fatiga - Dificultad para concentrarse - No hay fiebre, náuseas ni vómito |
| Historial médico pasado |  |
| Alergias a medicamentos (nombre y reacción) | Ninguna |
| Alergias ambientales (nombre y reacción) | Ninguna |
| Enfermedades | Ninguna |
| Medicaciones | Ninguna |
| Vacunas | Al día |
| Cirugias | Ninguna |
| Accidentes/lesiones/traumas | Ninguno |
| Hospitalización | Ninguna |
| Historial sexual y reproductivo inclusivo | |
| Prácticas sexuales  Parejas sexuales  Protección: uso de prácticas sexuales más seguras  Uso de anticonceptivos si corresponde  Riesgo de violencia por parte de la pareja íntima | Si se le pregunta:  - Es sexualmente activa con una pareja masculina desde hace 1 año  - No usa anticonceptivos hormonales  - No refiere infecciones previas  Si no se le pregunta:  - No ofrece esta información espontáneamente |
| Dieta (describa) | Dieta balanceada |
| Ejercicio (describa) | Corre 2 veces por semana |
| Enumere cualquier otro historial social importante o información relevante para este caso | Ninguna otra información relevante mencionada |
| Historial Familiar |  |
| Madre, padre, hermanos, abuelos y otros hallazgos significativos | - Madre: con antecedentes de depresión leve - Hermano: trastorno de ansiedad - Tía: endometriosis |

Acciones esperadas (críticas) del estudiante

**El estudiante debe:**

- Abrir el encuentro de manera respetuosa
- Elicitar la queja principal
- Explorar síntomas emocionales y físicos
- Identificar el patrón cíclico
- Preguntar sobre impacto funcional
- Utilizar lenguaje empático

**El estudiante debe evitar:**

- Minimizar los síntomas
- Hacer suposiciones
- Usar lenguaje técnico sin explicación

**Ejemplos de lenguaje esperado**

Apertura:

“Quiero asegurarme de que se sienta cómoda. Todo lo que hablemos es confidencial.”

Exploración:

“¿Cómo afectan estos síntomas su vida diaria o su trabajo?”

Empatía:

“Entiendo que esto puede ser muy difícil de manejar cada mes.”

Cierre:

“Vamos a explorar opciones para ayudarle a sentirse mejor.”

**Errores comunes**

- Enfocarse solo en síntomas físicos
- No explorar impacto emocional
- Uso de lenguaje directo sin empatía
- Cambiar al inglés innecesariamente

Guía de debriefing (para facilitadores)

**Preguntas sugeridas:**

- ¿Qué estrategias ayudaron a que el paciente se sintiera cómodo?
- ¿Cómo identificaron el patrón de los síntomas?
- ¿Qué lenguaje fue más difícil de usar?

**Puntos clave:**

- Validar la experiencia del paciente mejora la comunicación
- El patrón cíclico es esencial para el razonamiento clínico
- La claridad es más importante que la perfección gramatical

*- English -*

**Clinical Background for Facilitators**

*Evidence-based reference for facilitators and standardized patients. The learner-facing task is Spanish-language communication; this clinical content is provided for case fidelity and is not assessed.*

**Working diagnosis:** Premenstrual dysphoric disorder (PMDD); severe premenstrual syndrome (PMS).

**Chief complaint:** Recurrent emotional and physical symptoms in the luteal phase (days before menses) that resolve shortly after menstruation begins.

**Leading differential diagnoses:** PMDD/PMS; premenstrual exacerbation of an underlying mood or anxiety disorder; thyroid dysfunction.

**Evidence-based history:** Confirm cyclic timing (luteal-phase onset with resolution within a few days of menses); functional/occupational impact; mood and anxiety symptoms; thyroid symptoms; medications and substance use; psychiatric history.

**Examination:** No specific physical examination is required; the diagnosis is clinical and based on prospective symptom charting (vital signs as appropriate).

**Initial assessment/workup:** Prospective daily symptom diary across at least two menstrual cycles is the diagnostic standard; apply DSM-5 criteria for PMDD; consider TSH and depression/anxiety screening to exclude mimics.

**Reference:** Hofmeister S, Bodden S. Premenstrual Syndrome and Premenstrual Dysphoric Disorder. Am Fam Physician. 2016;94(3):236-240.

**Case Name:** Standardized Patient Case: Focused Reproductive Health Encounter with Severe emotional and physical symptoms prior to menstruation

**Encounter logistics**

**Type of encounter:** Formative standardized patient encounter

**Setting:** Outpatient

**Format:** Small-group (4 learners : 1 SP)

**Duration:** 25 minutes

**Learner task:** Focused reproductive history and patient-centered communication in Spanish

**Physical exam:** Not performed; verbalized only if applicable

**Counseling:** Not required

**Language requirement:** Spanish only

**Assessment:** Formative (not graded)

**Case-Specific Facilitation Notes**

This case is designed for small-group interaction (4 learners per SP) with assigned roles as described in Appendix A.

Facilitators should allow the interaction to proceed without interruption unless communication breaks down.

Focus observation on:

•	identification of cyclical symptom pattern

•	use of empathetic language

•	exploration of emotional and functional impact

This case emphasizes communication and pattern recognition rather than diagnostic precision.

**Formative Educational Activity**

**Methods**

Standardized Patients (SPs) received a detailed script in advance outlining the patient profile, including relevant personal characteristics and medical history, to ensure adequate familiarization with the case prior to the simulation.

The activity was conducted in the classroom. The clinical encounter took place at table-based stations, with an average of four students per table and one Standardized Patient, over an approximate period of 25 minutes.

Training materials included the detailed clinical case script.

| SETTING: outpatient, in patient, ED, home, nursing home, rehab, group, etc. | Outpatient |
| --- | --- |
| PATIENT PROFILE: Information about the “patient” that helps select an SP and helps the learner get an understanding of them as a person. SP will know more information about the patient than learner will ever ask but allows SP to portray a fully developed patient personality. If none of the items below are particulars for the case, please write “all may be used.” | |
| Patient’s name | Daniela Márquez |
| Pronouns | She/Her/Hers |
| Age range | 28 years old |
| Religious/spiritual background | Catholic, non-practicing |
| Sex (e.g., male, female, intersex, transwoman, transman) | Cisgender female |
| Sexual orientation (e.g., heterosexual, lesbian, gay, bisexual, pansexual, queer, asexual) | Straight |
| Gender expression (e.g., man, woman, genderqueer) | Woman |
| Race and/or ethnicity | Hispanic/Latinx |
| Physical description (e.g., BMI, height range) | Not specified, all may be used |
| Physical limitations | None |
| Patient appearance (e.g., disheveled, hospital gown, business casual, casual) | Casual, maintains eye contact but may sigh or appear overwhelmed when discussing symptoms |
| Moulage + location (e.g., none, bruises, scars, body piercing, tattoos) | None |
| Affect (e.g., pleasant, cooperative) | Cooperative, tearful and frustrated |
| The patient should: | Respond openly but not offer unsolicited information  Display a sad and frustrated affect, especially when describing emotional symptoms  Sigh or pause when discussing the impact on daily life  Provide more detail when the student uses empathetic language  Respond briefly if questions are direct or lack empathy  If the student uses unclear language:  Ask for clarification (“Can you repeat the question?”)  If the student switches to English:  Respond in Spanish and maintain the encounter in Spanish |
| Family group (e.g., who is family, who they live with) | Lives alone |
| Education | High school completed; currently works as a graphic designer |
| Level of health literacy | Moderate |
| Employment, if any - present and past, noting any current stresses | Graphic Designer |
| Home/homeless - type of dwelling, number of stories, owned or rented | Lives alone |
| Financial situation - any current stresses | Limited resources |
| Insurance status (e.g., un/under/insured, public/private, HMO/PPO) | Uninsured |
| Habits (i.e., diet, exercise, caffeine, smoking, alcohol, drugs) | Does not smoke or use recreational drugs; occasional alcohol use (1-2 times a month at social gatherings) |
| Activities (i.e., hobbies, sports, clubs, friends) | Likes to read and listen to music |
| Typical day - what is the usual daily routine | Works in person and virtually some days as a graphic designer |

| CASE INFORMATION | |
| --- | --- |
| Chief Concern: What the patient will say when greeted by the student. The patient’s primary reason for seeking medical care often stated in their own words. | “I’m here because before my period starts, I feel awful. It affects me a lot.” |
| Information Disclosure Rules | Initial information (without prompting):  - General symptoms prior to menstruation  Only if the student asks specifically:  Details of the menstrual cycle (duration, regularity)  - Impact on work   - Family history of mental health - Sexual activity and contraceptive use   If the student does not ask:  -Do not offer details about sexual or family history |
| Additional Concerns: Other, if any, concerns the patient has today (i.e., symptoms, requests, expectations, etc.) that will become part of set agenda. | “Every month, a few days before my period starts, I get super irritable. I cry over everything, I feel anxious, I don’t sleep well, and my body hurts. And then, once my period starts, everything goes away. I don’t know any more if this is normal.” |
| HISTORY OF PRESENT ILLNESS: Although some of the HPI will be given in the patient’s symptom story, the learners will expand the story during the direct question section. Below, describe the detailed history, usually about the chief concern, which the student must develop in order to make a useful assessment of the problem: | |
| Onset (when; gradual or sudden) | For approximately 8 months, but it has worsened over the last 3 cycles; gradual |
| Setting (what was going on or where was patient when symptoms first noticed?) | Gradual onset, no clear trigger |
| Duration (how long) | The symptoms appear 7 to 10 days before menstruation and disappear when bleeding starts |
| Time relationships (frequency, constant or intermittent) | Consistently for approximately 8 months |
| Location | Not applicable |
| Radiation | None |
| Quality | Moderate to severe. “Sometimes I cannot function well in my work” |
| Severity | Not applicable |
| Aggravated by what | Work stress and poor sleep make everything worse |
| Relieved by what | Nothing has worked well. Sometimes exercise helps a little |
| Associated with what | - Easy crying - Sudden mood changes - Insomnia - Intense irritability - Feeling guilty after an argument - Mild cramps - Fatigue - Difficulty concentrating - No fever, nausea, or vomiting |
| Past medical history |  |
| Medication allergies (name and reaction) | None |
| Environmental allergies (name and reaction) | None |
| Illnesses | None |
| Medications | None |
| Vaccinations | Up to date |
| Surgeries | None |
| Accidents/injuries/trauma | None |
| Hospitalization | None |
| Inclusive sexual and reproductive history | |
| Sexual practices  Sexual partners  Protection: Use of safer sex practices  Use of birth control if appropriate  Risk of intimate partner violence | If asked:   - She is sexually active with a male partner for the past 1 year - She does not use hormonal contraception - She does not report prior infections - If not asked: - She does not offer this information spontaneously |
| Diet (describe) | Balanced diet |
| Exercise (describe) | Runs two times a week |
| List any other important social history or information important to this case | No other relevant information mentioned |
| Family history |  |
| Mother, father, siblings, grandparents, and other significant findings | - Mother: History of mild depression - Brother: Generalized Anxiety Disorder - Aunt: Endometriosis |

Expected (Critical) Learner Actions

**The learner should:**

•	Open the encounter respectfully

•	Elicit the chief complaint

•	Explore both emotional and physical symptoms

•	Identify the cyclical pattern

•	Ask about functional impact

•	Use empathetic language

**The learner should avoid:**

•	Minimizing symptoms

•	Making assumptions

•	Using technical language without explanation

**Examples of Expected Language**

Opening:

“I want to make sure you feel comfortable. Everything we discuss is confidential.”

Exploration:

“How do these symptoms affect your daily life or your work?”

Empathy:

“I understand that this can be very difficult to manage each month.”

Closing:

“We are going to explore options to help you feel better.”

**Common Errors**

•	Focusing only on physical symptoms

•	Not exploring emotional impact

•	Using direct language without empathy

•	Switching to English unnecessarily

Debriefing Guide (for facilitators)

**Suggested questions:**

•	What strategies helped the patient feel comfortable?

•	How did you identify the pattern of symptoms?

•	What language was most difficult to use?

**Key points:**

•	Validating the patient’s experience improves communication

•	The cyclical pattern is essential for clinical reasoning

•	Clarity is more important than grammatical perfection
