# Supplementary material for: Reproductive Health Communication in Spanish: A Standardized Patient-Based Module for Second-Year Medical Students
Source: MedEdPORTAL. 2026 Sep 8;22:11635. doi: 10.15766/mep_2374-8265.11635 (PMC13550226; doi:10.15766/mep_2374-8265.11635)
Supplement: Supplementary file 1 [file mep_2374-8265.11635-s001.zip › F. SP Case Lesson 2.docx]

**Appendix F.** Standardized Patient Case: Lesson 2 (Pregnancy-Related Concerns)

Thought process

**Contexto clínico para facilitadores**
*Referencia basada en evidencia para facilitadores y pacientes estandarizados. La tarea del estudiante es la comunicación en español; este contenido clínico se proporciona para la fidelidad del caso y no se evalúa.*

**Diagnóstico de trabajo:** Embarazo con hipertensión crónica, con riesgo de preeclampsia sobreañadida.

**Motivo de consulta:** Consulta prenatal con posibles síntomas de alarma de preeclampsia (cefalea persistente, visión borrosa, hinchazón).

**Diagnósticos diferenciales principales:** Hipertensión crónica en el embarazo; preeclampsia sobreañadida (con o sin datos de gravedad); hipertensión gestacional.

**Anamnesis basada en evidencia:** Edad gestacional; presión arterial basal y medicamentos antihipertensivos/adherencia; signos de alarma de preeclampsia (cefalea intensa o persistente, alteraciones visuales, dolor en el cuadrante superior derecho o epigástrico, hinchazón de aparición rápida, disminución de los movimientos fetales); antecedentes obstétricos y médicos.

**Exploración física:** Presión arterial (clave); valoración de edema; (verbalizado) altura uterina y frecuencia cardíaca fetal según corresponda.

**Evaluación/estudio inicial:** Mediciones seriadas de la presión arterial; proteinuria (índice proteína:creatinina); panel de laboratorio para preeclampsia (hemograma, creatinina, AST/ALT, +/- ácido úrico); valoración fetal; evaluar preeclampsia con datos de gravedad según ACOG.

**Referencia:** American College of Obstetricians and Gynecologists. Gestational Hypertension and Preeclampsia. ACOG Practice Bulletin No. 222. Obstet Gynecol. 2020;135(6):e237-e260.

**Nombre del caso:** Caso de paciente estandarizado: evaluación reproductiva con embarazo con hipertensión crónica (riesgo de preeclampsia)

**Tipo de encuentro:** Encuentro formativo con paciente estandarizado

**Escenario:** Ambulatorio

Formato: Grupo pequeño (4 estudiantes : 1 paciente estandarizado)

**Duración:** 25 minutos

**Tarea del estudiante:** Historia clínica reproductiva enfocada y comunicación centrada en el paciente en español

**Examen físico**: No se realiza; solo se verbaliza si corresponde

**Consejería:** No requerida

**Idioma requerido:** Solo español

**Evaluación:** Formativa (no calificada)

**Notas de facilitación específicas del caso**

Este caso está diseñado para la interacción en grupos pequeños (4 estudiantes por paciente estandarizado), con roles asignados según se describe en el Apéndice A.

Los facilitadores deben permitir que la interacción se desarrolle sin interrupciones, a menos que la comunicación se deteriore.

Enfoque de observación:

• identificación de signos de alarma en el embarazo (cefalea persistente, visión borrosa, edema)

• exploración dirigida de antecedentes relevantes (hipertensión, medicación, evolución del embarazo)

• uso de lenguaje empático ante la preocupación por el bienestar del bebé

• organización lógica de la historia clínica

Este caso enfatiza la comunicación clínica y el reconocimiento de factores de riesgo en el embarazo más que la precisión diagnóstica.

**Actividad Educacional Formativa**

**Métodos**

Los Pacientes Estandarizados (PE) recibieron previamente un guion detallado con el perfil del paciente, así como información sobre las características personales y el historial médico relevante, con el fin de familiarizarse adecuadamente con el caso antes de la simulación.

La actividad se llevó a cabo en el salón de clase. El encuentro clínico se desarrolló en mesas de trabajo, con un promedio de cuatro estudiantes por mesa y un Paciente Estandarizado, durante un período aproximado de 25 minutos.

Los materiales de capacitación incluyeron el guión clínico detallado del caso.

| ESCENARIO: Ambulatorio, hospitalizado, urgencias, hogar, residencia de ancianos, rehabilitación, grupo, etc. | Ambulatorio |
| --- | --- |
| PERFIL DEL PACIENTE: Información sobre el “paciente” que ayuda a seleccionar un SP y ayuda al aprendiz a comprenderlos como persona. El SP conocerá más información sobre el paciente de la que el aprendiz preguntará, pero permite que el SP represente una personalidad de paciente completamente desarrollada. Si ninguno de los elementos a continuación es particular para el caso, escriba “se pueden usar todos”. | |
| Nombre del paciente | Laura Martínez |
| Pronombre | Ella |
| Rango de edad | 37 años |
| Religión/ antecedentes espirituales | Cristiano/a |
| Sexo (masculino, femenino, intersexual, transgénero…) | Mujer cisgénero |
| Orientación sexual (por ejemplo, heterosexual, lesbiana, gay, bisexual, pansexual, queer, asexual) | Heterosexual (actual pareja masculina) |
| Expresión de género (por ejemplo, hombre, mujer, género no binario) | Mujer |
| Raza y/o etnia | Hispana/Latinx |
| Descripción física (por ejemplo, IMC, rango de altura) | Embarazo de 30 semanas de gestación (índice de masa corporal ligeramente elevado; estatura promedio) |
| Limitaciones físicas | Ninguna; refiere edema leve en las extremidades inferiores al final del día. |
| Apariencia del paciente (por ejemplo, desaliñado, bata de hospital, informal de negocios) | Informal; limpia y apropiada para una consulta prenatal. |
| Simulación + ubicación (por ejemplo, ninguna, moretones, cicatrices, piercings, tatuajes) | Ninguna |
| Afecto (por ejemplo, agradable, cooperativo) | Cooperativa, ligeramente ansiosa |
| El paciente debe: | Responder de manera abierta pero no ofrecer información no solicitada  Mostrar afecto ansioso y preocupado, especialmente al hablar de los síntomas y del bienestar del bebé  Expresar preocupación cuando menciona la presión arterial alta o los cambios recientes en su salud  Responder con más detalle cuando el estudiante usa lenguaje empático  Responder de forma breve si las preguntas son directas o poco empáticas  Si el estudiante usa lenguaje poco claro:  Pedir aclaración (“¿Puede repetir la pregunta?”)  Si el estudiante cambia a inglés:  Responder en español y mantener el encuentro en español |
| Grupo familiar (por ejemplo, quiénes son familiares, con quién viven) | Vive con su esposo y su hijo |
| Educación | Universidad incompleta |
| Nivel de alfabetización en salud | Moderado |
| Empleo, si lo hay: presente y pasado, señalando cualquier estrés actual | Empleada administrativa; trabajo sedentario; refiere estrés laboral relacionado con el embarazo y preocupaciones de salud. |
| Hogar/sin hogar - tipo de vivienda, número de pisos, propiedad o alquilada | Vive con su familia |
| Situación financiera - cualquier estrés actual | Generalmente estable; algunas preocupaciones relacionadas con los costos de atención médica y las necesidades asociadas al embarazo. |
| Estado del seguro (por ejemplo, no asegurado/infrasegurado/asegurado, público/privado, HMO/PPO) | Con seguro médico |
| Hábitos (es decir, dieta, ejercicio, cafeína, fumar, alcohol, drogas) | Dieta regular; reconoce que consume sal en exceso; no fuma, usa drogas recreativas, o consume alcohol. |
| Actividades (es decir, pasatiempos, deportes, clubes, amigos) | Pasa tiempo con su familia; actividad física limitada debido al embarazo y la fatiga. |
| Día típico - cuál es la rutina diaria habitual | Trabaja durante el día en un entorno de oficina; regresa a casa por la tarde para cuidar de su familia; descansa cuando es posible debido a la fatiga y la hinchazón. |

| CASE INFORMATION | |
| --- | --- |
| Queja principal: Lo que el paciente dirá cuando sea recibido por el estudiante. La razón principal del paciente para buscar atención médica, a menudo expresada en sus propias palabras | “He tenido dolor de cabeza y se me han hinchado los pies.” |
| Reglas de divulgación de información | Información inicial (sin que el estudiante pregunte):  - Dolor de cabeza persistente  - Hinchazón en los pies  - Embarazo de aproximadamente 30 semanas  Solo si el estudiante pregunta específicamente:  - Episodios de visión borrosa  - Antecedente de hipertensión arterial  - Medicación actual (labetalol)  - Evolución reciente del embarazo  - Impacto de los síntomas en su vida diaria  - Preocupación por el bienestar del bebé  Si el estudiante no pregunta:  - No ofrecer detalles sobre antecedentes médicos, medicamentos, ni síntomas asociados adicionales |
| Preocupaciones adicionales: Otras preocupaciones que el paciente tenga hoy (es decir, síntomas, solicitudes, expectativas, etc.) que se incluirán en la agenda establecida | Episodios ocasionales de visión borrosa, preocupación por presión arterial alta mencionada en la última visita prenatal, y miedo de que esto pueda afectar al bebé. |
| HISTORIA DE LA ENFERMEDAD ACTUAL: Aunque parte de la historia de la enfermedad actual se dará en el relato de los síntomas del paciente, los aprendices ampliarán la historia durante la sección de preguntas directas. A continuación, describa la historia detallada, generalmente sobre la queja principal, que el estudiante debe desarrollar para hacer una evaluación útil del problema: | |
| Inicio (cuándo; gradual o repentino) | Hace aproximadamente 1 semana |
| Contexto (qué estaba pasando o dónde estaba el paciente cuando se notaron los síntomas por primera vez) | Inicio gradual; Durante su rutina diaria y notado en casa y después del trabajo |
| Duración (cuánto tiempo) | Persistente desde hace una semana |
| Relaciones de tiempo (frecuencia, constante o intermitente) | - Dolor de cabeza: casi constante - Visión borrosa: intermitente |
| Ubicación | Dolor de cabeza: Frente y sienes |
| Radiación | Ninguna |
| Calidad | Opresivo, como presión |
| Intensidad | No especificado |
| Empeorado por qué | Levantarse rápido; al final del día |
| Aliviado por qué | Acetaminofén ayuda parcialmente, pero no lo quita por completo |
| Asociado con qué | - Presión arterial elevada - Hinchazón en pies - Visión borrosa ocasional |
| Historial médico pasado |  |
| Alergias a medicamentos (nombre y reacción) | Niega alergias medicamentosas |
| Alergias ambientales (nombre y reacción) | Ninguna |
| Enfermedades | Hipertensión arterial crónica desde hace 5 años - previamente controlada antes del embarazo; con elevaciones recientes |
| Medicaciones | - Hipertensión: Labetalol 200 mg dos veces al día - Dolor de cabeza: Acetaminofén ocasional - Vitaminas prenatales |
| Vacunas | Al día |
| Cirugías | Ninguna |
| Accidentes/lesiones/traumas | Ninguno |
| Hospitalización | Solo por parto previo |
| Historial sexual y reproductivo inclusivo | |
| Prácticas sexuales  Parejas sexuales  Protección: uso de prácticas sexuales más seguras  Uso de anticonceptivos si corresponde  Riesgo de violencia por parte de la pareja íntima | Si se le pregunta:  - Es sexualmente activa con su esposo  - Niega infecciones de transmisión sexual previas  - Niega sangrado vaginal o pérdida de líquido  - Niega riesgo de violencia por parte de la pareja íntima  Si no se le pregunta:  - No ofrece esta información espontáneamente |
| Dieta (describa) | Dieta regular pero reconoce que consume sal en exceso |
| Ejercicio (describa) | No especificado |
| Enumere cualquier otro historial social importante o información relevante para este caso | Ninguna otra información relevante mencionada |
| Historial Familiar |  |
| Madre, padre, hermanos, abuelos y otros hallazgos significativos | - Madre: hipertensión - Padre: diabetes tipo 2 - Otros: niega antecedentes familiares de preeclampsia |

Acciones esperadas (críticas) del estudiante

**El estudiante debe:**

- Abrir el encuentro de manera respetuosa
- Elicitar la queja principal
- Explorar síntomas relevantes del embarazo (cefalea, visión borrosa, edema)
- Identificar signos de alarma en el embarazo
- Preguntar sobre antecedentes médicos relevantes (hipertensión, medicación, evolución del embarazo)
- Evaluar el impacto funcional de los síntomas
- Utilizar lenguaje empático

**El estudiante debe evitar:**

- Minimizar los síntomas
- Hacer suposiciones
- Usar lenguaje técnico sin explicación

**Ejemplos de lenguaje esperado**

Apertura:

“Quiero asegurarme de que se sienta cómoda. Todo lo que hablemos es confidencial.”

Exploración:

“¿Ha notado cambios en su visión o hinchazón reciente?”

“¿Desde cuándo ha tenido estos síntomas?”

Empatía:

“Entiendo que esto puede ser preocupante, especialmente durante el embarazo.”

Cierre:

“Vamos a evaluar estos síntomas para asegurarnos de que usted y su bebé estén bien.”

**Errores comunes**

- No explorar síntomas clave del embarazo
- No identificar signos de alarma
- Uso de lenguaje directo sin empatía
- Cambiar al inglés innecesariamente

Guía de debriefing (para facilitadores)

**Preguntas sugeridas:**

- ¿Qué signos de alarma identificaron?
- ¿Qué preguntas ayudaron a aclarar el riesgo?
- ¿Qué lenguaje fue más difícil de usar?

**Puntos clave:**

- La identificación de signos de alarma es esencial en el embarazo
- El lenguaje empático mejora la comunicación
- La claridad es más importante que la perfección gramatical

*- English -*

**Clinical Background for Facilitators**

*Evidence-based reference for facilitators and standardized patients. The learner-facing task is Spanish-language communication; this clinical content is provided for case fidelity and is not assessed.*

**Working diagnosis:** Pregnancy with chronic hypertension, at risk for superimposed preeclampsia.

**Chief complaint:** Prenatal visit with possible preeclampsia warning symptoms (persistent headache, blurred vision, swelling).

**Leading differential diagnoses:** Chronic hypertension in pregnancy; superimposed preeclampsia (with or without severe features); gestational hypertension.

**Evidence-based history:** Gestational age; baseline blood pressure and antihypertensive medications/adherence; preeclampsia warning signs (severe or persistent headache, visual changes, right-upper-quadrant or epigastric pain, rapid swelling, decreased fetal movement); obstetric and medical history.

**Examination:** Blood pressure (key); assessment for edema; (verbalized) fundal height and fetal heart tones as appropriate.

**Initial assessment/workup:** Serial blood pressures; urine protein (protein:creatinine ratio); preeclampsia laboratory panel (CBC, creatinine, AST/ALT, +/- uric acid); fetal assessment; evaluate for preeclampsia with severe features per ACOG.

**Reference:** American College of Obstetricians and Gynecologists. Gestational Hypertension and Preeclampsia. ACOG Practice Bulletin No. 222. Obstet Gynecol. 2020;135(6):e237-e260.

**Case Name:** Standardized Patient Case: Focused Reproductive Health Encounter with Pregnancy with Chronic Hypertension (Risk of Preeclampsia)

**Encounter logistics**

**Type of encounter:** Formative standardized patient encounter

**Setting:** Outpatient

**Format:** Small-group (4 learners : 1 SP)

**Duration:** 25 minutes

**Learner task:** Focused reproductive history and patient-centered communication in Spanish

**Physical exam:** Not performed; verbalized only if applicable

**Counseling:** Not required

**Language requirement:** Spanish only

**Assessment:** Formative (not graded)

**Case-Specific Facilitation Notes**

This case is designed for small-group interaction (4 learners per standardized patient), with roles assigned as described in Appendix A.

Facilitators should allow the interaction to proceed without interruption unless communication breaks down.

Focus of observation:

• identification of warning signs in pregnancy (persistent headache, blurred vision, edema)

• targeted exploration of relevant history (hypertension, medications, course of pregnancy)

• use of empathetic language in response to concern about the baby’s well-being

• logical organization of the clinical history

This case emphasizes clinical communication and recognition of pregnancy-related risk factors rather than diagnostic precision.

**Formative Educational Activity**

**Methods**

Standardized Patients (SPs) received a detailed script in advance outlining the patient profile, including relevant personal characteristics and medical history, to ensure adequate familiarization with the case prior to the simulation.

The activity was conducted in the classroom. The clinical encounter took place at table-based stations, with an average of four students per table and one Standardized Patient, over an approximate period of 25 minutes.

Training materials included the detailed clinical case script.

| SETTING: outpatient, in patient, ED, home, nursing home, rehab, group, etc. | Outpatient |
| --- | --- |
| PATIENT PROFILE: Information about the “patient” that helps select an SP and helps the learner get an understanding of them as a person. SP will know more information about the patient than learner will ever ask but allows SP to portray a fully developed patient personality. If none of the items below are particulars for the case, please write “all may be used.” | |
| Patient’s name | Laura Martínez |
| Pronouns | She/Her/Hers |
| Age range | 37 years old |
| Religious/spiritual background | Christian |
| Sex (e.g., male, female, intersex, transwoman, transman) | Cisgender female |
| Sexual orientation (e.g., heterosexual, lesbian, gay, bisexual, pansexual, queer, asexual) | Straight (current partner: male) |
| Gender expression (e.g., man, woman, genderqueer) | Woman |
| Race and/or ethnicity | Hispanic/Latinx |
| Physical description (e.g., BMI, height range) | Pregnant at 30 week’s gestation; BMI mildly elevated; average height |
| Physical limitations | None; notes mild lower extremity swelling by end of day |
| Patient appearance (e.g., disheveled, hospital gown, business casual, casual) | Casual, clean, appropriate for prenatal visit |
| Moulage + location (e.g., none, bruises, scars, body piercing, tattoos) | None |
| Affect (e.g., pleasant, cooperative) | Cooperative, mildly anxious |
| The patient should: | Respond openly but do not offer unsolicited information  Display an anxious and concerned affect, especially when discussing symptoms and the baby’s well-being  Express concern when mentioning high blood pressure or recent changes in health  Provide more detail when the student uses empathetic language  Respond briefly if questions are direct or lack empathy  If the student uses unclear language:  Ask for clarification (“Could you repeat the question?”)  If the student switches to English:  Respond in Spanish and maintain the encounter in Spanish |
| Family group (e.g., who is family, who they live with) | Lives with husband and child |
| Education | Some college (university incomplete) |
| Level of health literacy | Moderate |
| Employment, if any - present and past, noting any current stresses | Administrative employee; sedentary work; reports work-related stress due to pregnancy and health concerns |
| Home/homeless - type of dwelling, number of stories, owned or rented | Lives with family |
| Financial situation - any current stresses | Generally stable; some concerns about healthcare cost and pregnancy-related needs |
| Insurance status (e.g., un/under/insured, public/private, HMO/PPO) | Insured |
| Habits (i.e., diet, exercise, caffeine, smoking, alcohol, drugs) | Diet regular; acknowledges high salt intake; does not smoke, use recreational drugs, or drink alcohol |
| Activities (i.e., hobbies, sports, clubs, friends) | Spends time with family; limited exercise due to pregnancy and fatigue |
| Typical day - what is the usual daily routine | Works during the day in an office setting, returns home in the evening to care for family; rests when possible due to fatigue and swelling |

| CASE INFORMATION | |
| --- | --- |
| Chief Concern: What the patient will say when greeted by the student. The patient’s primary reason for seeking medical care often stated in their own words. | “I've had a headache and my feet are swollen.” |
| Information disclosure rules | Initial information (without the student asking): persistent headache, swelling in the feet, and a pregnancy of approximately 30 weeks.  Only if the student asks specifically: episodes of blurred vision, history of hypertension, current medication (labetalol), recent progression of the pregnancy, impact of symptoms on daily life, and concern about the baby’s well-being.  If the student does not ask: do not offer details about medical history, medications, or additional associated symptoms. |
| Additional Concerns: Other, if any, concerns the patient has today (i.e., symptoms, requests, expectations, etc.) that will become part of set agenda. | Occasional episodes of blurred vision, concern about high blood pressure mentioned at the last prenatal visit, and fear that this could affect the baby |
| HISTORY OF PRESENT ILLNESS: Although some of the HPI will be given in the patient’s symptom story, the learners will expand the story during the direct question section. Below, describe the detailed history, usually about the chief concern, which the student must develop in order to make a useful assessment of the problem: | |
| Onset (when; gradual or sudden) | Approximately 1 week ago |
| Setting (what was going on or where was patient when symptoms first noticed?) | Gradual onset; during their daily routine and noticed at home and after work |
| Duration (how long) | For the past week |
| Time relationships (frequency, constant or intermittent) | - Headache: almost constant - Blurred vision: intermittent |
| Location | Headache: Forehead and temples |
| Radiation | None |
| Quality | Dull, pressure-like |
| Severity | Not specified |
| Aggravated by what | Getting up quickly; at the end of the day |
| Relieved by what | Acetaminophen helps, but doesn't completely eliminate it |
| Associated with what | - Elevated blood pressure - Swelling in the feet - Occasional blurred vision |
| Past medical history |  |
| Medication allergies (name and reaction) | Denies medication allergies |
| Environmental allergies (name and reaction) | None |
| Illnesses | Chronic hypertension for the past 5 years – previously controlled before pregnancy; with recent elevations |
| Medications | - Hypertension: Labetalol 200 mg twice daily - Headache: Acetaminophen occasionally - Prenatal vitamins |
| Vaccinations | Up to date |
| Surgeries | None |
| Accidents/injuries/trauma | None |
| Hospitalization | Only related to prior delivery |
| Inclusive sexual and reproductive history | |
| Sexual practices  Sexual partners  Protection: Use of safer sex practices  Use of birth control if appropriate  Risk of intimate partner violence | If asked:  - She is sexually active with her husband  - Denies prior sexually transmitted infections  - Denies vaginal bleeding or loss of fluid  - Denies risk of intimate partner violence  If not asked:  - Does not offer this information spontaneously |
| Diet (describe) | Regular diet but acknowledges excessive salt consumption |
| Exercise (describe) | Not specified |
| List any other important social history or information important to this case | No other relevant information mentioned |
| Family history |  |
| Mother, father, siblings, grandparents, and other significant findings | - Mother: Hypertension - Father: Type 2 Diabetes - Other: Denies family history of preeclampsia |

Expected (Critical) Learner Actions

**The student should:**

- Open the encounter respectfully
- Elicit the chief complaint
- Explore pregnancy-related symptoms (headache, blurred vision, edema)
- Identify warning signs in pregnancy
- Ask about relevant medical history (hypertension, medications, course of pregnancy)
- Assess the functional impact of symptoms
- Use empathetic language

**The student should avoid:**

- Minimizing symptoms
- Making assumptions
- Using technical language without explanation

**Examples of Expected Language**

Opening:

“I want to make sure you feel comfortable. Everything we discuss is confidential.”

Exploration:

“Have you noticed any changes in your vision or recent swelling?”

“How long have you been experiencing these symptoms?”

Empathy:

“I understand this can be concerning, especially during pregnancy.”

Closing:

“We are going to evaluate these symptoms to make sure that you and your baby are doing well.”

**Common Errors**

- Not exploring key pregnancy-related symptoms
- Not identifying warning signs
- Using direct language without empathy
- Switching to English unnecessarily

Debriefing Guide (for facilitators)

**Suggested questions:**

- What warning signs did you identify?
- What questions helped clarify the level of risk?
- What language was most difficult to use?

**Key points:**

- Identification of warning signs is essential in pregnancy
- Empathetic language improves communication
- Clarity is more important than grammatical perfection
