# Supplementary material for: Reproductive Health Communication in Spanish: A Standardized Patient-Based Module for Second-Year Medical Students
Source: MedEdPORTAL. 2026 Sep 8;22:11635. doi: 10.15766/mep_2374-8265.11635 (PMC13550226; doi:10.15766/mep_2374-8265.11635)
Supplement: Supplementary file 1 [file mep_2374-8265.11635-s001.zip › G. SP Case Lesson 3.docx]

**Appendix G.** Standardized Patient Case: Lesson 3 (Reproductive Examination Concerns)

**Contexto clínico para facilitadores**
Referencia basada en evidencia para facilitadores y pacientes estandarizados. La tarea del estudiante es la comunicación en español; este contenido clínico se proporciona para la fidelidad del caso y no se evalúa.

**Diagnóstico de trabajo:** Dolor abdominal bajo agudo no diferenciado en una mujer en edad reproductiva (baja gravedad; causas gastrointestinales/funcionales frente a causas ginecológicas).

**Motivo de consulta:** Dolor abdominal bajo de inicio reciente e intermitente, con estreñimiento, distensión abdominal y disminución del apetito; sin fiebre ni síntomas urinarios.

**Diagnósticos diferenciales principales:** Causas ginecológicas que no deben pasarse por alto y que hay que descartar (embarazo ectópico, enfermedad inflamatoria pélvica, rotura/torsión de quiste ovárico); causas gastrointestinales/funcionales (estreñimiento, dolor funcional); apendicitis; infección de las vías urinarias.

**Anamnesis basada en evidencia:** Caracterizar el dolor (p. ej., SOCRATES); fecha de la última menstruación y patrón menstrual; antecedentes sexuales y anticonceptivos; riesgo de infecciones de transmisión sexual; flujo o sangrado vaginal; síntomas gastrointestinales y genitourinarios; dieta y ejercicio.

**Exploración física:** Signos vitales; exploración abdominal; exploración pélvica (con espéculo y bimanual, incluyendo dolor a la movilización cervical) — solo verbalizada.

**Evaluación/estudio inicial:** Obtener primero una prueba de hCG en orina o suero en toda mujer en edad reproductiva con dolor pélvico; análisis de orina; hemograma; pruebas de ITS/NAAT; ecografía transvaginal (+/- transabdominal) como estudio de imagen de primera línea cuando se sospecha una causa ginecológica.

**Referencia:** Bhavsar AK, Gelner EJ, Shorma T. Common Questions About the Evaluation of Acute Pelvic Pain. Am Fam Physician. 2016;93(1):41-48.

**Nombre del caso:** Evaluación de salud reproductiva centrada en dolor abdominal bajo en una persona adulta sin seguro

**Tipo de encuentro:** Encuentro formativo con paciente estandarizado

**Escenario:** Ambulatorio

Formato: Grupo pequeño (4 estudiantes : 1 paciente estandarizado)

**Duración:** 25 minutos

**Tarea del estudiante:** Historia clínica reproductiva enfocada y comunicación centrada en el paciente en español

**Examen físico**: No se realiza; solo se verbaliza si corresponde

**Consejería:** No requerida

**Idioma requerido:** Solo español

**Evaluación:** Formativa (no calificada)

**Notas de facilitación específicas del caso**

Este caso está diseñado para la interacción en grupos pequeños (4 estudiantes por paciente estandarizado), con roles asignados según se describe en el Apéndice A.

Los facilitadores deben permitir que la interacción se desarrolle sin interrupciones, a menos que la comunicación se deteriore.

Enfoque de observación:

- identificación de características del dolor abdominal (localización, inicio, intensidad, síntomas asociados)
- exploración de síntomas gastrointestinales y genitourinarios relevantes
- evaluación del impacto funcional
- uso de lenguaje empático
- organización lógica de la historia clínica

Este caso enfatiza la comunicación clínica y el reconocimiento de factores de riesgo en el embarazo más que la precisión diagnóstica.

**Actividad Educacional Formativa**

**Métodos**

Los Pacientes Estandarizados (PE) recibieron previamente un guion detallado con el perfil del paciente, así como información sobre las características personales y el historial médico relevante, con el fin de familiarizarse adecuadamente con el caso antes de la simulación.

La actividad se llevó a cabo en el salón de clase. El encuentro clínico se desarrolló en mesas de trabajo, con un promedio de cuatro estudiantes por mesa y un Paciente Estandarizado, durante un período aproximado de 25 minutos.

Los materiales de capacitación incluyeron el guión clínico detallado del caso.

| ESCENARIO: Ambulatorio, hospitalizado, urgencias, hogar, residencia de ancianos, rehabilitación, grupo, etc. | Ambulatorio |
| --- | --- |
| PERFIL DEL PACIENTE: Información sobre el “paciente” que ayuda a seleccionar un SP y ayuda al aprendiz a comprenderlos como persona. El SP conocerá más información sobre el paciente de la que el aprendiz preguntará, pero permite que el SP represente una personalidad de paciente completamente desarrollada. Si ninguno de los elementos a continuación es particular para el caso, escriba “se pueden usar todos”. | |
| Nombre del paciente | Alejandro Montes |
| Pronombre | Él |
| Rango de edad | 39 años |
| Religión/ antecedentes espirituales | Cristiano/a |
| Sexo (masculino, femenino, intersexual, transgénero…) | Hombre cisgénero |
| Orientación sexual (por ejemplo, heterosexual, lesbiana, gay, bisexual, pansexual, queer, asexual) | Gay |
| Expresión de género (por ejemplo, hombre, mujer, género no binario) | Hombre |
| Raza y/o etnia | Hispano/Latinx |
| Descripción física (por ejemplo, IMC, rango de altura) | 168 cm de altura y 85 kg de peso |
| Limitaciones físicas | Ninguna |
| Apariencia del paciente (por ejemplo, desaliñado, bata de hospital, informal de negocios) | Informal; ligeramente incómodo/a por el dolor abdominal |
| Simulación + ubicación (por ejemplo, ninguna, moretones, cicatrices, piercings, tatuajes) | Ninguna |
| Afecto (por ejemplo, agradable, cooperativo) | Cooperativo, ligeramente ansioso |
| El paciente debe: | Responder de manera abierta pero no ofrecer información no solicitada  Mostrar incomodidad leve por el dolor abdominal, con momentos de molestia al describir los síntomas  Expresar preocupación sobre la causa del dolor y su acceso limitado a atención médica  Responder con más detalle cuando el estudiante usa lenguaje empático  Responder de forma breve si las preguntas son directas o poco empáticas  Si el estudiante usa lenguaje poco claro:  Pedir aclaración (“¿Puede repetir la pregunta?”)  Si el estudiante cambia a inglés:  Responder en español y mantener el encuentro en español |
| Grupo familiar (por ejemplo, quiénes son familiares, con quién viven) | Vive con su madre y un hermano menor |
| Educación | Escuela secundaria completada; actualmente estudiante universitario de primer año |
| Nivel de alfabetización en salud | Moderado |
| Empleo, si lo hay: presente y pasado, señalando cualquier estrés actual | Estudiante a tiempo completo; no trabaja |
| Hogar/sin hogar - tipo de vivienda, número de pisos, propiedad o alquilada | Vive con su familia |
| Situación financiera - cualquier estrés actual | Recursos limitados |
| Estado del seguro (por ejemplo, no asegurado/infrasegurado/asegurado, público/privado, HMO/PPO) | Sin seguro médico |
| Hábitos (es decir, dieta, ejercicio, cafeína, fumar, alcohol, drogas) | No fuma ni usa drogas recreativas; consumo ocasional de alcohol (2–3 bebidas en reuniones) |
| Actividades (es decir, pasatiempos, deportes, clubes, amigos) | Estudios universitarios |
| Día típico - cuál es la rutina diaria habitual | Asiste a clases y estudia en casa |

| CASE INFORMATION | |
| --- | --- |
| Queja principal: Lo que el paciente dirá cuando sea recibido por el estudiante. La razón principal del paciente para buscar atención médica, a menudo expresada en sus propias palabras | “Me duele la parte baja del abdomen desde hace unos días.” |
| Reglas de divulgación de información | Información inicial (sin que el estudiante pregunte):  Dolor abdominal bajo  Inicio reciente del dolor  Molestia intermitente  Solo si el estudiante pregunta específicamente:  Localización exacta del dolor  Intensidad y características del dolor  Factores que empeoran o alivian el dolor  Síntomas asociados (náuseas, fiebre, cambios urinarios o gastrointestinales si aplica)  Impacto en la vida diaria  Acceso limitado a atención médica  Si el estudiante no pregunta:  No ofrecer detalles adicionales sobre síntomas asociados ni antecedentes relevantes |
| Preocupaciones adicionales: Otras preocupaciones que el paciente tenga hoy (es decir, síntomas, solicitudes, expectativas, etc.) que se incluirán en la agenda establecida | Le preocupa la causa del dolor y su acceso limitado a servicios médicos por falta de seguro. |
| HISTORIA DE LA ENFERMEDAD ACTUAL: Aunque parte de la historia de la enfermedad actual se dará en el relato de los síntomas del paciente, los aprendices ampliarán la historia durante la sección de preguntas directas. A continuación, describa la historia detallada, generalmente sobre la queja principal, que el estudiante debe desarrollar para hacer una evaluación útil del problema: | |
| Inicio (cuándo; gradual o repentino) | Hace 4 días |
| Contexto (qué estaba pasando o dónde estaba el paciente cuando se notaron los síntomas por primera vez) | Inicio gradual, sin desencadenante claro |
| Duración (cuánto tiempo) | Persistente durante varios días |
| Relaciones de tiempo (frecuencia, constante o intermitente) | Empeora al final del día |
| Ubicación | Parte baja del abdomen, especialmente el lado izquierdo |
| Radiación | Ninguna |
| Calidad | Dolor cólico con momentos de punzadas |
| Intensidad | 6/10 |
| Empeorado por qué | Estar sentado por períodos prolongados |
| Aliviado por qué | Descanso |
| Asociado con qué | - No fiebre, vómitos ni diarrea - No náuseas, pero con disminución del apetito - Orina con normalidad - Estreñimiento (última evacuación hace 2 días) - Hinchazón occasional - Niega síntomas urinarios |
| Historial médico pasado |  |
| Alergias a medicamentos (nombre y reacción) | Penicilina (erupción leve) |
| Alergias ambientales (nombre y reacción) | Ninguna |
| Enfermedades | Ninguna |
| Medicaciones | Ninguna |
| Vacunas | Al día |
| Cirugias | Ninguna |
| Accidentes/lesiones/traumas | Ninguno |
| Hospitalización | Ninguna |
| Historial sexual y reproductivo inclusivo | |
| Prácticas sexuales  Parejas sexuales  Protección: uso de prácticas sexuales más seguras  Uso de anticonceptivos si corresponde  Riesgo de violencia por parte de la pareja íntima | - Activo sexualmente - Múltiples parejas sexuales - Protección: uso de prácticas sexuales más seguras - Uso de anticonceptivos si corresponde - Riesgo de violencia por parte de la pareja íntima |
| Dieta (describa) | Dieta balanceada pero alta en carbohidratos |
| Ejercicio (describa) | Gimnasio tres veces a la semana |
| Enumere cualquier otro historial social importante o información relevante para este caso | Ninguna otra información relevante mencionada |
| Historial Familiar |  |
| Madre, padre, hermanos, abuelos y otros hallazgos significativos | - Madre: quistes ováricos - Padre: hiperplasia prostática benigna - Tía materna: cáncer de cuello uterino - Abuelos: diabetes tipo 2 e hipertensión |

Acciones esperadas (críticas) del estudiante

**El estudiante debe:**

•	Abrir el encuentro de manera respetuosa

•	Elicitar la queja principal

•	Explorar las características del dolor abdominal (localización, inicio, intensidad, síntomas asociados)

•	Preguntar sobre síntomas gastrointestinales y genitourinarios relevantes

•	Evaluar el impacto funcional

•	Utilizar lenguaje empático

**El estudiante debe evitar:**

•	Minimizar los síntomas

•	Hacer suposiciones

•	Usar lenguaje técnico sin explicación

**Ejemplos de lenguaje esperado**

Apertura:

“Quiero asegurarme de que se sienta cómodo. Todo lo que hablemos es confidencial.”

Exploración:

“¿Puede describir dónde está el dolor y cuándo empezó?”

“¿Este dolor ha afectado sus actividades diarias?”

Empatía:

“Entiendo que esto puede ser preocupante.”

Cierre:

“Vamos a evaluar sus síntomas para entender mejor qué está causando este dolor.”

**Errores comunes**

**•**	Evaluación incompleta del dolor

•	No explorar síntomas asociados

•	Uso de lenguaje directo sin empatía

•	Cambiar al inglés innecesariamente

Guía de debriefing (para facilitadores)

**Preguntas sugeridas:**

•	¿Qué preguntas ayudaron a aclarar los síntomas del paciente?

•	¿Cómo estructuraron la evaluación del dolor?

•	¿Qué lenguaje fue más difícil de usar?

**Puntos clave:**

•	Una evaluación estructurada del dolor apoya el razonamiento clínico

•	El lenguaje empático mejora la comunicación con el paciente

•	La claridad es más importante que la perfección gramatical

*- English -*

**Clinical Background for Facilitators**

*Evidence-based reference for facilitators and standardized patients. The learner-facing task is Spanish-language communication; this clinical content is provided for case fidelity and is not assessed.*

**Working diagnosis:** Undifferentiated acute lower abdominal pain in a reproductive-age woman (low-acuity; gastrointestinal/functional vs. gynecologic causes).

**Chief complaint:** Recent-onset, intermittent lower abdominal pain with constipation, bloating, and decreased appetite; no fever or urinary symptoms.

**Leading differential diagnoses:** Can't-miss gynecologic causes to exclude (ectopic pregnancy, pelvic inflammatory disease, ovarian cyst rupture/torsion); gastrointestinal/functional causes (constipation, functional pain); appendicitis; urinary tract infection.

**Evidence-based history:** Characterize the pain (e.g., SOCRATES); last menstrual period and menstrual pattern; sexual and contraceptive history; sexually transmitted infection risk; vaginal discharge or bleeding; gastrointestinal and genitourinary symptoms; diet and exercise.

**Examination:** Vital signs; abdominal examination; pelvic examination (speculum and bimanual, including cervical motion tenderness) - verbalized only.

**Initial assessment/workup:** Obtain a urine or serum hCG first in any reproductive-age woman with pelvic pain; urinalysis; CBC; STI/NAAT testing; transvaginal (+/- transabdominal) ultrasound as first-line imaging when a gynecologic cause is suspected.

**Reference:** Bhavsar AK, Gelner EJ, Shorma T. Common Questions About the Evaluation of Acute Pelvic Pain. Am Fam Physician. 2016;93(1):41-48.

**Case Name:** Focused Reproductive Health Evaluation for Lower Abdominal Pain in an Uninsured Adult

**Type of encounter:** Formative standardized patient encounter

**Setting:** Outpatient

**Format:** Small group (4 learners : 1 standardized patient)

**Duration:** 25 minutes

**Learner task:** Focused reproductive history and patient-centered communication in Spanish

**Physical exam:** Not performed; verbalized only if applicable

**Counseling:** Not required

**Language requirement:** Spanish only

**Assessment:** Formative (not graded)

**Case-specific facilitation notes**

This case is designed for small-group interaction (4 learners per standardized patient), with assigned roles as described in Appendix A.

Facilitators should allow the interaction to proceed without interruption unless communication breaks down.

**Focus of observation:**

- identification of abdominal pain characteristics (location, onset, severity, associated symptoms)
- exploration of relevant gastrointestinal and genitourinary symptoms
- assessment of functional impact
- use of empathetic language
- logical organization of the clinical history

This case emphasizes clinical communication and recognition of pregnancy risk factors rather than diagnostic precision.

**Formative Educational Activity**

**Methods**

Standardized Patients (SPs) received a detailed script in advance outlining the patient profile, including relevant personal characteristics and medical history, to ensure adequate familiarization with the case prior to the simulation.

The activity was conducted in the classroom. The clinical encounter took place at table-based stations, with an average of four students per table and one Standardized Patient, over an approximate period of 25 minutes.

Training materials included the detailed clinical case script.

| SETTING: outpatient, in patient, ED, home, nursing home, rehab, group, etc. | Outpatient |
| --- | --- |
| PATIENT PROFILE: Information about the “patient” that helps select an SP and helps the learner get an understanding of them as a person. SP will know more information about the patient than learner will ever ask but allows SP to portray a fully developed patient personality. If none of the items below are particulars for the case, please write “all may be used.” | |
| Patient’s name | Alejandro Montes |
| Pronouns | He/Him/his |
| Age range | 39 years old |
| Religious/spiritual background | Christian |
| Sex (e.g., male, female, intersex, transwoman, transman) | Cisgender male |
| Sexual orientation (e.g., heterosexual, lesbian, gay, bisexual, pansexual, queer, asexual) | Gay |
| Gender expression (e.g., man, woman, genderqueer) | Man |
| Race and/or ethnicity | Hispanic/Latinx |
| Physical description (e.g., BMI, height range) | 168 cm in height and 85 kg in weight |
| Physical limitations | None |
| Patient appearance (e.g., disheveled, hospital gown, business casual, casual) | Casual, mildly uncomfortable due to abdominal pain |
| Moulage + location (e.g., none, bruises, scars, body piercing, tattoos) | None |
| Affect (e.g., pleasant, cooperative) | Cooperative, mildly anxious |
| The patient should: | Respond openly but do not offer unsolicited information  Show mild discomfort due to abdominal pain, with moments of irritation when describing symptoms  Express concern about the cause of the pain and limited access to medical care  Provide more detailed responses when the student uses empathetic language  Respond briefly if questions are direct or lack empathy  If the student uses unclear language:  Ask for clarification (“Can you repeat the question?”)  If the student switches to English:  Respond in Spanish and maintain the encounter in Spanish |
| Family group (e.g., who is family, who they live with) | Lives with mother and younger sibling |
| Education | High school completed; currently a first-year university student |
| Level of health literacy | Moderate |
| Employment, if any - present and past, noting any current stresses | Full-time student; does not work |
| Home/homeless - type of dwelling, number of stories, owned or rented | Lives with family |
| Financial situation - any current stresses | Limited resources |
| Insurance status (e.g., un/under/insured, public/private, HMO/PPO) | Uninsured |
| Habits (i.e., diet, exercise, caffeine, smoking, alcohol, drugs) | Does not smoke or use recreational drugs; occasional alcohol use (2–3 drinks at social gatherings) |
| Activities (i.e., hobbies, sports, clubs, friends) | University studies |
| Typical day - what is the usual daily routine | Attends classes and studies at home |

| CASE INFORMATION | |
| --- | --- |
| Chief Concern: What the patient will say when greeted by the student. The patient’s primary reason for seeking medical care often stated in their own words. | “My lower abdomen has been hurting for the past few days.” |
| Information Disclosure Rules | Initial information (without the student asking):  Lower abdominal pain  Recent onset of pain  Intermittent discomfort  Only if the student asks specifically:  Exact location of the pain  Intensity and characteristics of the pain  Factors that worsen or relieve the pain  Associated symptoms (nausea, fever, urinary or gastrointestinal changes if applicable)  Impact on daily life  Limited access to medical care  If the student does not ask:  Do not offer additional details about associated symptoms or relevant medical history |
| Additional Concerns: Other, if any, concerns the patient has today (i.e., symptoms, requests, expectations, etc.) that will become part of set agenda. | The patient is concerned about the cause of the pain and has limited access to medical care due to lack of insurance. |
| HISTORY OF PRESENT ILLNESS: Although some of the HPI will be given in the patient’s symptom story, the learners will expand the story during the direct question section. Below, describe the detailed history, usually about the chief concern, which the student must develop in order to make a useful assessment of the problem: | |
| Onset (when; gradual or sudden) | 4 days ago |
| Setting (what was going on or where was patient when symptoms first noticed?) | Gradual onset, no clear trigger |
| Duration (how long) | Ongoing over several days |
| Time relationships (frequency, constant or intermittent) | Worse at the end of the day |
| Location | Lower abdomen, especially the left side |
| Radiation | None |
| Quality | Cramping pain with intermittent sharp sensations |
| Severity | 6/10 |
| Aggravated by what | Prolonged sitting |
| Relieved by what | Rest |
| Associated with what | - No fever, vomiting, or diarrhea - No nausea, but decreased appetite - Normal urination - Constipation (last bowel movement 2 days ago) - Occasional bloating - No abnormal urinary symptoms |
| Past medical history |  |
| Medication allergies (name and reaction) | Penicillin (mild rash) |
| Environmental allergies (name and reaction) | None |
| Illnesses | None |
| Medications | None |
| Vaccinations | Up to date |
| Surgeries | None |
| Accidents/injuries/trauma | None |
| Hospitalization | None |
| Inclusive sexual and reproductive history | |
| Sexual practices  Sexual partners  Protection: Use of safer sex practices  Use of birth control if appropriate  Risk of intimate partner violence | - Active sexually - Multiple partners - Protection: use of safer sexual practices - Use of contraception if applicable - Risk of intimate partner violence |
| Diet (describe) | Balanced diet but high in carbohydrates |
| Exercise (describe) | Gym three times a week |
| List any other important social history or information important to this case | No other relevant information mentioned |
| Family history |  |
| Mother, father, siblings, grandparents, and other significant findings | - Mother: Ovarian cysts - Father: Benign prostatic hyperplasia - Maternal aunt: Cervical cancer - Grandparents: Type 2 diabetes and hypertension |

Expected (Critical) Learner Actions

**The learner should:**

•	Open the encounter respectfully

•	Elicit the chief complaint

•	Explore characteristics of abdominal pain (location, onset, severity, associated symptoms)

•	Ask about relevant gastrointestinal and genitourinary symptoms

•	Assess functional impact

•	Use empathetic language

**The learner should avoid:**

•	Minimizing symptoms

•	Making assumptions

•	Using technical language without explanation

**Examples of Expected Language**

Opening:

“I want to make sure you feel comfortable. Everything we discuss is confidential.”

Exploration:

“Can you describe where the pain is and when it started?”

“Has this pain affected your daily activities?”

Empathy:

“I understand that this can be concerning.”

Closing:

“We are going to evaluate your symptoms to better understand what is causing this pain.”

Common Errors

•	Incomplete pain assessment

•	Not exploring associated symptoms

•	Using direct language without empathy

•	Switching to English unnecessarily

Debriefing Guide (for facilitators)

**Suggested questions:**

•	What questions helped clarify the patient’s symptoms?

•	How did you structure your pain assessment?

•	What language was most difficult to use?

**Key points:**

•	A structured pain assessment supports clinical reasoning

•	Empathetic language improves patient communication

•	Clarity is more important than grammatical perfection
