# Supplementary material for: Reproductive Health Communication in Spanish: A Standardized Patient-Based Module for Second-Year Medical Students
Source: MedEdPORTAL. 2026 Sep 8;22:11635. doi: 10.15766/mep_2374-8265.11635 (PMC13550226; doi:10.15766/mep_2374-8265.11635)
Supplement: Supplementary file 1 [file mep_2374-8265.11635-s001.zip › J. SP Recruitment.docx]

**Appendix J.** Standardized Patient Recruitment, Training, Portrayal, and Feedback Procedures

**1. Standardized Patient Recruitment Criteria**

Standardized Patients (SPs) were recruited through institutional hiring processes within the Department of Medical Education at the California University of Science and Medicine (CUSM) for part-time, non-exempt positions.

Eligibility criteria included:

•	Advanced oral proficiency in Spanish and functional proficiency in English

•	High school diploma or equivalent

•	Ability to accurately and consistently portray a patient role

•	Strong interpersonal and communication skills

•	Ability to provide structured, objective feedback to learners

•	Comfort participating in simulated clinical encounters, including observation and video recording for educational purposes

Preferred qualifications included:

•	Prior experience as a standardized patient

•	Familiarity with medical terminology

•	Experience in teaching or tutoring environments

•	Intermediate written proficiency in Spanish

SPs were required to demonstrate the ability to:

•	Memorize and reproduce case scripts accurately

•	Maintain standardization across multiple learner encounters

•	Interact professionally with learners from diverse backgrounds

•	Provide consistent portrayal without introducing personal bias or variability

**2. Training Procedures**

SP training followed a structured, multi-step process designed to ensure consistency and reproducibility across encounters.

Pre-training preparation:

•	SPs received the complete case script in advance

•	SPs were compensated for a dedicated independent study period to review and memorize the case content

Structured training sessions:

•	SPs participated in a formal training session led by the Standardized Patient Educator

•	During this session:

•	The case was reviewed in detail

•	Clinical content, emotional tone, and response patterns were clarified

•	Expected learner behaviors and prompts were discussed

•	Standardization expectations were emphasized

•	SPs were encouraged to ask questions and clarify uncertainties

On-site calibration and rehearsal:

•	SPs attended an additional in-person training session prior to implementation

•	This session focused on:

•	Reinforcing consistency across SPs

•	Practicing portrayal and timing

•	Aligning responses to ensure uniform delivery of case information

•	Clarifying any remaining uncertainties

**3. Portrayal Instructions and Standardization**

SPs were trained to provide consistent, controlled, and standardized portrayals across all learner encounters.

Portrayal expectations included:

•	Responding accurately according to the case script

•	Avoiding unsolicited information unless specified in disclosure rules

•	Maintaining consistent emotional affect as described in the case

•	Modulating responses based on learner communication style (e.g., providing more detail when empathy is demonstrated)

Standardization rules:

•	Provide only information requested by the learner unless otherwise specified

•	Maintain consistency in wording, tone, and level of detail across encounters

•	Request clarification if learner communication is unclear

•	Maintain the encounter in Spanish when required

Behavioral calibration:

SPs were instructed to:

•	Provide more detailed responses when learners use empathetic, patient-centered language

•	Provide brief or limited responses when questions are direct, unclear, or lack empathy

•	Reflect emotional and functional impact as described in the case

**4. Session Oversight and Quality Assurance**

During instructional sessions:

•	The Standardized Patient Educator was present to observe SP performance

•	Real-time monitoring ensured:

•	adherence to the case script

•	consistency across SPs

•	appropriate use of expected terminology and tone

•	Immediate clarification was provided if deviations from standardization were observed

This process supported consistency across learner experiences and minimized variability in SP portrayal.

**5. Feedback Procedures**

Following each encounter, SPs provided brief, structured feedback to learners from the patient perspective, focused on clarity of communication, comprehensibility of the learner’s Spanish, and interpersonal communication, using the Standardized Patient Structured Feedback Checklist provided at the end of this appendix. This feedback was formative and was not scored or used for grading.

Faculty facilitators provided additional feedback to learners during the post-encounter debrief.

SPs were also monitored and provided with feedback by the Standardized Patient Educator on an individual basis to ensure accurate and consistent portrayal.

SP performance feedback focused on:

•	Adherence to the case script

•	Consistency of responses across encounters

•	Appropriate emotional affect and tone

•	Alignment with expected communication patterns

Quality assurance and calibration:

SP portrayal was reviewed during training sessions and, when necessary, during live sessions. Individualized feedback was provided to SPs to address deviations and reinforce standardization across encounters.

**6. Alignment with Educational Objectives**

SP recruitment, training, and portrayal procedures were designed to support:

•	Standardization across learner experiences

•	Reliable evaluation of communication skills

•	Reproducibility of the educational intervention

This structured approach ensured that SP performance remained consistent and aligned with the communication-focused objectives of the curriculum.

**Standardized Patient Structured Feedback Checklist**

Completed by the standardized patient immediately after each encounter to guide brief verbal feedback to the learner. The checklist is formative and is not scored or used for grading. Mark each item Yes, Somewhat, or Not yet.

| **Item** | **Yes** | **Somewhat** | **Not yet** |
| --- | --- | --- | --- |
| 1. Greeted me and introduced themselves. |  |  |  |
| 2. Made me feel comfortable and respected. |  |  |  |
| 3. Showed attention to comfort and privacy when discussing sensitive topics. |  |  |  |
| 4. Used respectful, inclusive, non-assumptive language. |  |  |  |
| 5. Spoke Spanish I could understand. |  |  |  |
| 6. Explained things clearly and checked my understanding. |  |  |  |
| 7. Asked about my main concern (chief complaint). |  |  |  |
| 8. Asked relevant follow-up questions about my symptoms. |  |  |  |

**One thing you did well:** _______________________________________________

**One suggestion to improve:** _______________________________________________
