# Supplementary material for: Reproductive Health Communication in Spanish: A Standardized Patient-Based Module for Second-Year Medical Students
Source: MedEdPORTAL. 2026 Sep 8;22:11635. doi: 10.15766/mep_2374-8265.11635 (PMC13550226; doi:10.15766/mep_2374-8265.11635)
Supplement: Supplementary file 1 [file mep_2374-8265.11635-s001.zip › K. POLOM Rating Tool Scoring Guide.docx]

**Appendix K.** POLOM Rating Tool, Scoring Guide, and Rater Orientation

**1. Assessment Framework**

Learner performance during the OSCE was evaluated using the Physician Oral Language Observation Matrix (POLOM), an observational rating instrument developed and validated to assess medical oral language proficiency among medical students during Spanish-language standardized patient encounters.

The POLOM evaluates six domains of language performance:

•	Comprehension

•	Fluency

•	Vocabulary

•	Pronunciation

•	Grammar

•	Communication (patient-centered interaction and task completion)

Each domain reflects functional communicative ability within a clinical encounter.

**2. Scoring Scale**

Each domain was rated using a 5-point ordinal scale, where:

•	Level 1 = Unable to communicate effectively; communication breakdown

•	Level 2 = Significant limitations; communication frequently impaired

•	Level 3 = Basic functional communication; some strain but meaning preserved

•	Level 4 = Effective communication with minor limitations

•	Level 5 = Clear, accurate, and patient-centered communication without difficulty

Scores were assigned based on overall performance across the encounter rather than isolated errors.

**3. Domain Interpretation for This Study**

Raters were instructed to evaluate each domain as follows:

•	Comprehension: Ability to understand patient responses and respond appropriately

•	Fluency: Continuity and flow of speech without excessive pauses

•	Vocabulary: Use of appropriate clinical and general language, including ability to explain terms

•	Pronunciation: Clarity and intelligibility of speech

•	Grammar: Accuracy of sentence structure and verb use, with emphasis on meaning

•	Communication: Ability to build rapport, use empathy, adjust language appropriately, and conduct a patient-centered encounter

**4. Scoring Principles**

Raters were trained to prioritize:

•	Overall communicative effectiveness over grammatical perfection

•	Patient comprehension and comfort

•	Organization and clarity of the clinical encounter

Errors were considered significant only if they:

•	impeded understanding

•	disrupted the flow of the interaction

•	negatively affected patient-centered communication

**5. Rater Training Procedures**

Evaluations were conducted by faculty with experience in medical Spanish instruction and clinical communication.

Rater orientation was delivered as a single live, synchronous virtual (videoconference) session of approximately 60 minutes prior to the OSCE.

Rater preparation included:

•	Review of POLOM domains and scoring criteria

•	Discussion of performance expectations aligned with course objectives

•	Calibration through review of sample performances and scoring scenarios

•	Emphasis on consistent application of scoring standards across learners

Raters were instructed to:

•	Evaluate each domain independently

•	Base scores on the full encounter rather than isolated moments

•	Avoid over-penalizing minor linguistic errors

**6. Standardization and Reliability**

To support consistency:

•	Raters followed a shared scoring framework based on POLOM definitions

•	Scoring emphasized observable behaviors and communication outcomes

•	Calibration discussions were used to align scoring expectations prior to evaluation

**7. Sample Calibration Performances (De-identified)**

The following synthetic, de-identified samples illustrate performance at varying POLOM proficiency levels and were reviewed during rater calibration to align scoring. They are illustrative and do not reproduce any actual learner encounter. Each sample includes a brief learner–standardized patient excerpt in Spanish followed by a domain-by-domain rationale mapped to the POLOM level descriptors (1–5). Domains are scored independently.

**Sample 1 — Emerging proficiency**

*Learner: “Hola. Yo soy estudiante. Eh… ¿qué problema… tener usted? ¿Dónde… dolor?”*

*Standardized patient: “Me duele aquí abajo, desde hace unos días.”*

*Learner: “Eh… ¿desde… cuándo?… um, sorry… ¿mucho dolor?”*

**Comprehension — 3:** Understood the patient only when she answered slowly and repeated “desde hace unos días”; repetition was required, but no misunderstanding altered the clinical content (Level 3: understands at slower-than-normal speed or with frequent repetitions).

**Fluency — 2:** Hesitant for most of the encounter, with frequent pauses (“eh…,” “um…”) and a shift into English (“sorry”) that strained the conversation (Level 2: hesitant most of the time; forced into English or unnatural pauses).

**Vocabulary — 2:** Used inadequate, insufficient vocabulary and needed English; could not produce terms to characterize the symptom (Level 2: inadequate vocabulary most of the time and needing to use English).

**Pronunciation — 3:** Generally intelligible but required some concentration from the rater at points, occasionally straining the exchange (Level 3: requires some concentration and/or sometimes strains the conversation).

**Grammar — 2:** Restricted to basic patterns with errors that strained comprehension — an uninflected verb (“¿qué problema tener?”) and verbless questions (“¿Dónde dolor?”) (Level 2: too often restricted to basic patterns; errors make some sentences difficult).

**Communication — 2:** Opened with a basic greeting and self-introduction but did not establish comfort, privacy, or confidentiality, and the language limitations impeded the flow of the interview (Level 2: basic greetings, but limitations impede communication at some point).

**Sample 2 — Functional proficiency**

*Learner: “Buenos días, soy estudiante de medicina. ¿Me puede decir qué la trae hoy?”*

*Standardized patient: “Tengo un dolor en la parte baja del abdomen desde hace tres días.”*

*Learner: “Entiendo. ¿Cuándo empezó exactamente y cómo es el dolor? ¿Es… cómo se dice… constante o va y viene?”*

*Standardized patient: “Va y viene.”*

*Learner: “De acuerdo. ¿Ha tenido náuseas o cambios en la… ‘bowel’… en sus evacuaciones?”*

**Comprehension — 4:** Understood the history at normal conversational speed, with no repetition required and no misunderstanding (Level 4: understands nearly everything at normal speed).

**Fluency — 3:** Allowed the conversation to proceed but was disrupted by an occasional lapse while searching for expression (“…cómo se dice…”) that she eventually resolved (Level 3: lapses due to the search for expression but eventually able to express it).

**Vocabulary — 4:** Briefly reached for an English medical term (“bowel”) but immediately supplied the correct Spanish equivalent (“evacuaciones”), so content was unaffected (Level 4: occasional English medical term but easily provides a clear explanation).

**Pronunciation — 4:** Consistently intelligible, with only occasional intonation that did not cause misunderstanding or strain (Level 4: consistently intelligible).

**Grammar — 3:** Used structures beyond basic patterns but made occasional errors that briefly strained a sentence while remaining understandable (Level 3: errors that strain the conversation at some point yet can be understood).

**Communication — 4:** Introduced herself, used open and encouraging questions, and acknowledged the patient (“Entiendo”), adjusting register and showing appropriate empathy with only occasional difficulty (Level 4: shows empathy appropriately; few limitations).

**Sample 3 — Proficient**

*Learner: “Buenos días, mi nombre es [nombre], soy estudiante de medicina. Antes de comenzar, quiero asegurarme de que se sienta cómoda; todo lo que hablemos es confidencial. ¿Podría contarme qué la trae hoy?”*

*Standardized patient: “Tengo dolor en la parte baja del vientre.”*

*Learner: “Lamento que se sienta así. ¿Cuándo comenzó el dolor, dónde lo siente exactamente y cómo lo describiría? ¿Hay algo que lo mejore o lo empeore?”*

**Comprehension — 5:** Understood the patient at normal speed throughout, with no need for repetition (Level 5: understands at normal speed without difficulty).

**Fluency — 5:** Speech was fluid, smooth, and effortless (Level 5).

**Vocabulary — 5:** Used precise vocabulary and appropriate lay terms (“parte baja del vientre”), avoiding jargon (Level 5: wide variety of vocabulary; avoids technical jargon as needed).

**Pronunciation — 5:** Pronunciation was completely clear (Level 5: totally clear).

**Grammar — 5:** Grammar and word order were consistently accurate (Level 5).

**Communication — 5:** Built strong rapport, ensured confidentiality and comfort, showed empathy (“Lamento que se sienta así”), organized the interview, and addressed sensitive content appropriately with no difficulty (Level 5).

**8. Use of the POLOM Instrument**

The POLOM was used as the underlying framework for evaluation. The full instrument is described in the original publication:

Diamond LC, Gregorich SE, Karliner L, et al. Development of a Tool to Assess Medical Oral Language Proficiency. Acad Med. 2023;98(4):480-490. doi:10.1097/ACM.0000000000004942
