# Supplementary material for: Reproductive Health Communication in Spanish: A Standardized Patient-Based Module for Second-Year Medical Students
Source: MedEdPORTAL. 2026 Sep 8;22:11635. doi: 10.15766/mep_2374-8265.11635 (PMC13550226; doi:10.15766/mep_2374-8265.11635)
Supplement: Supplementary file 1 [file mep_2374-8265.11635-s001.zip › M. Door Note and learner instructions.docx]

**Appendix M.** Door Note and Learner Instructions

**OSCE Door Note**

**Station Title:**

Reproductive Health Encounter: Lower Abdominal Pain

**Setting:**

Outpatient clinic

**Time:**

12 minutes

**Patient Name:** Adriana García

**Age:** 29 years

**Chief Complaint:**

“My lower abdomen has been hurting for the past few days.”

**Instructions:**

You are a medical student in an outpatient clinic. Your task is to conduct a focused clinical encounter in Spanish.

You should:

•	Elicit the chief complaint

•	Obtain a focused history of present illness

•	Clarify symptom characteristics (e.g., onset, location, severity, associated symptoms)

•	Obtain relevant reproductive, menstrual, and sexual history

•	Ask appropriate family and social history questions

•	Use patient-centered communication, including empathy and respectful language

Important:

•	Conduct the encounter entirely in Spanish

•	Do NOT perform a physical exam (you may verbalize if appropriate)

•	Do NOT provide counseling or diagnosis

Your goal is to demonstrate:

•	Clear and organized clinical communication in Spanish

•	Appropriate use of reproductive health vocabulary

•	Ability to obtain a focused reproductive and sexual health history

•	Patient-centered communication, including empathy, respect, and non-assumptive language

You are expected to:

•	Introduce yourself and establish rapport

•	Ensure patient comfort and confidentiality

•	Elicit the chief complaint

•	Explore the history of present illness using a structured approach (e.g., SOCRATES when appropriate)

•	Ask relevant reproductive, menstrual, and sexual history questions

•	Assess impact on daily life

•	Use clear, simple, and respectful language

You should avoid:

•	Using English unless absolutely necessary

•	Making assumptions about the patient

•	Using technical language without explanation

•	Providing diagnosis or counseling
