# Supplementary material for: Reproductive Health Communication in Spanish: A Standardized Patient-Based Module for Second-Year Medical Students
Source: MedEdPORTAL. 2026 Sep 8;22:11635. doi: 10.15766/mep_2374-8265.11635 (PMC13550226; doi:10.15766/mep_2374-8265.11635)
Supplement: Supplementary file 1 [file mep_2374-8265.11635-s001.zip › N. End-of-Module Evaluation Survey.docx]

**Appendix N.** End-of-Module Evaluation Survey

**Instructions to Students**

Please rate the following statements based on your experience in this module.

Use the scale below:

1 = Strongly Disagree

2 = Disagree

3 = Agree

4 = Strongly Agree

Likert-Scale Items

Module Structure and Organization

1.	The module was well organized.

2.	The sequence of sessions supported my learning.

3.	The content was appropriate for my level of Spanish proficiency.

Learning Environment

4.	The learning environment was supportive and engaging.

5.	I felt comfortable participating in activities and speaking Spanish.

Opportunities for Practice

6.	The module provided sufficient opportunities to practice clinical Spanish.

7.	The standardized patient encounters supported my learning.

8.	The small-group activities helped reinforce key concepts.

Clinical Relevance

9.	The content was relevant to clinical practice.

10.	The module helped me understand how to approach reproductive and sexual health conversations in Spanish.

Perceived Learning Outcomes

11.	This module improved my ability to communicate with patients in Spanish.

12.	This module improved my confidence in using Spanish in clinical settings.

13.	I am better able to obtain a focused reproductive and sexual health history in Spanish.

Optional Open-Ended Questions

14.	What aspects of the module were most helpful for your learning?

15.	What could be improved in this module?

16.	Any additional comments:
